# Emerging computational and machine learning methodologies for proton-conducting oxides: materials discovery and fundamental understanding

**DOI:** 10.1080/14686996.2024.2416383

**Published:** 2024-10-29

**Authors:** Susumu Fujii, Junji Hyodo, Kazuki Shitara, Akihide Kuwabara, Shusuke Kasamatsu, Yoshihiro Yamazaki

**Affiliations:** a Department of Materials, Faculty of Engineering, Kyushu University, Fukuok, Japan; b Nanostructures Research Laboratory, Japan Fine Ceramics Center, Nagoya, Japan; c Center for Energy System Design (CESD), International Institute for Carbon Neutral Energy Research (WPI-I2CNER), Kyushu University, Fukuoka, Japan; d Faculty of Science, Yamagata University, Yamagata, Japan; e Kyushu University Platform of Inter-/Transdisciplinary Energy Research (Q-PIT), Kyushu University, Fukuoka, Japan

**Keywords:** Proton-conducting oxides, first-principles calculation, machine learning, hydration, proton diffusion, materials discovery through interpretation

## Abstract

This review presents computational and machine learning methodologies developed during a 5-year research project on proton-conducting oxides. The main goal was to develop methodologies that could assist in materials discovery or provide new insights into complex proton-conducting oxides. Through these methodologies, three new proton-conducting oxides, including both perovskite and non-perovskites, have been discovered. In terms of gaining insights, octahedral tilt/distortions and oxygen affinity are found to play a critical role in determining proton diffusivities and conductivities in doped barium zirconates. Replica exchange Monte Carlo approach has enabled to reveal realistic defect configurations, hydration behavior, and their temperature dependence in oxides. Our approach ‘Materials discovery through interpretation’, which integrates new insights or tendencies obtained from computations and experiments to sequential explorations of materials, has also identified perovskites that exhibit proton conductivity exceeding 0.01 S/cm and high chemical stability at 300  ∘C.

## IMPACT STATEMENT

This review presents new computational and machine learning methodologies that identified three new proton-conducting oxides and two fast proton conductors. It also provides new insights into hydration, proton diffusion, and proton conduction.

## GRAPHICAL ABSTRACT

**Figure:**
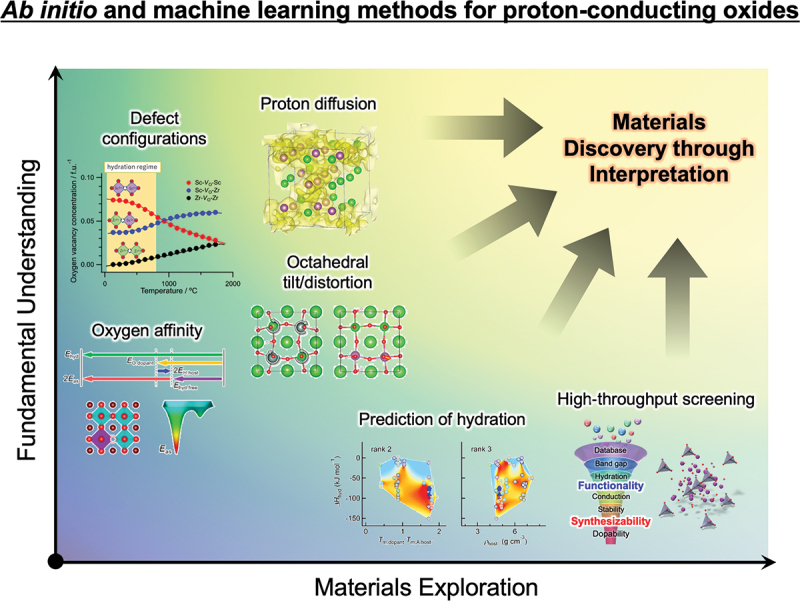

## 1. Introduction

Computational materials science, particularly *ab initio* calculation, has become the *de facto* standard approach utilized in materials research [1,2]. Its role encompasses not only obtaining microscopic interpretation of material functions but also searching for novel materials through high-throughput calculations using modern supercomputers. The former role has traditionally been to reveal the origin of superior material functions discovered experimentally, inspiring researchers and helping the optimization of material functions. The latter role, on the other hand, replaces the main part of material exploration with computation rather than experiment, and aims to significantly accelerate the exploration by only synthesizing and measuring promising candidates suggested by high-throughput screening. *Ab initio* calculations for inorganic solids in the ideal defect-free state have become routine tasks, as is clear from the well-established *ab initio* databases, such as Materials Project [3] and AFlow [4]. Currently, gaining fundamental insights of *ab initio* calculations and performing material screening has become much easier by using these databases and associated tools. Furthermore, the enormous amount of material data generated by *ab initio* calculations has also enabled the development of machine learning (ML) models to predict structures, chemical stabilities, and physical properties of materials, making the analysis and exploration increasingly fast [5,6].

In the case of inorganic solid materials, however, it is common that lattice defects activate material functions even with a small amount of defects incorporated in the materials [7–11]. This is true for a wide spectrum of materials, including solar cells [8], catalysts [9], batteries [10], fuel cells [11], and so on. Among these, point defects are ubiquitous defects that even exist in single crystals and often have decisive effects on macroscopic material properties. These include vacancies, interstitials, and impurity atoms (dopants). Their concentration and behavior are significantly dependent on the chemistry of materials and equilibrium with solid and gas phases [12]. In other words, sample composition, partial pressures of gas phases such as O2 and H2O, temperature and pressure, all affect the defect chemistry. In addition, the point defects may exist in neutral and charged states and compete with electronic defects such as holes, making the situation more complex. *Ab initio* calculations are suitable approach for interpreting the behavior of point defects at the nanoscale, but defect calculations require much higher computational costs than bulk crystals, limiting their applications to simplified cases. Developing new approaches for efficiently handling these point defects and their interactions as in Ref. [13] is vital in the fields of solid-state chemistry and physics.

Proton-conducting oxides are prime examples where point defects play a key role in determining their functionality [14–17] and are the subject of this review. They can serve as solid electrolytes in which interstitial protons are charge carriers, being important for the realization of environmentally friendly electrochemical devices including solid oxide fuel cells and electrolyzers. Historically, doped SrCeO3 perovskite was the first proton-conducting oxide discovered by Iwahara et al. in 1981 [18], and the perovskites such as BaCeO3- and BaZrO3-based systems have remained at the main focus of the research and development of proton-conducting oxides [14,16]. Proton conduction in oxides is the result of successive reactions involving point defects [14,16]. In short, acceptor dopants are added to a host oxide to create oxygen vacancies, and then the water uptake through the vacancy sites (hydration) creates two hydroxyl groups OH− or interstitial protons H+, and finally the diffusion of interstitial protons results in the appearance of conductivity (Section 2). The choice of host-dopant combination and their ratio simply determine whether or not these reactions and diffusion occur, but the underlying phenomena behind them, *i.e*. interaction and competition between several types of point-defects and holes, makes the interpretation difficult. Many experimental efforts have been devoted to revealing the hydration thermodynamics, proton conductivity and diffusivity, and their governing factors (Sections 3-4). These have successfully showed the critical roles of point defect interactions on proton conduction, *i.e*. dopant-vacancy association, dopant-proton association, and vacancy-hole competition on proton concentration and diffusivity.

Computational studies have been performed for proton-conducting oxides in line with the most advanced measurements of the time [19–23]. *Ab initio* calculations for point defects have quantified the energies of dopant-vacancy and dopant-proton associations, which are highly dependent on the chemistry of the host and dopant, and also their effects on hydration thermodynamics and proton diffusion (Section 5.1). Molecular dynamics (MD) simulations have also shown a Grotthus-like mechanism of proton diffusion, where protons rotate around an oxide-ion site and hop to another neighboring oxygen site (Section 5.2). Elucidation of this mechanism and calculated proton migration energy barrier and association energies have enabled kinetic Monte Carlo (kMC) simulations, which can evaluate long-time proton diffusion and reproduce the conductivity peak with respect to dopant concentration in Y-doped BaZrO3 (Section 5.2). As such, computational approaches based mainly on *ab initio* calculations are an important means of interpreting the microscopic behavior of point defects and suggesting optimization strategies for proton-conducting oxides based on the defect chemistry.

While the computational efforts in the past few decades have provided a fundamental interpretation of proton conduction, they have the drawback of simplifying the computational models and thus leave many questions unanswered for the behavior of point defects (Section 5). This is the complex interactions between the defects and associated structural changes that become more important at high dopant concentration. Most energy calculations to date considered only a few patterns for the possible configurations of oxygen vacancies, dopants, and protons at 0 K (Section 5.1). Also, most MD and kMC simulations assumed random configurations of dopants for estimating proton diffusivity (Section 5.2). There are, however, innumerable possible configurations of the point defects in reality, and the actual configurations depend on the association energy between defects as well as the configuration entropy at a finite temperature (Section 10). Recently, high proton concentration and diffusivity have been achieved in heavily doped materials such as BaZr0.4Sc0.6O3−δ [24], and thus the proton conduction cannot be fully understood without treating more realistic correlations and configurations of point defects (Sections 9-10). In addition, point defects are known to significantly change the local structures of host materials (Section 3.2). Such defect–lattice interactions should be more carefully considered, as it has a non-negligible impact on the formation and association energies on point defects as well as proton diffusion (Section 8).

There are also challenges in terms of computational material exploration. First, most of the above experimental and computational findings on proton-conducting oxides are for perovskites, and our basic understanding of non-perovskite oxides [25] is limited. Even the simplified point-defect calculations, which consider only a few patterns of defect configurations, have scarcely been performed for non-perovskite oxides, hindering material exploration in a wider structural and compositional space (Section 6). In addition, few examples of data-driven materials discovery, where the stability and functionality of a target compound were experimentally demonstrated, have been reported, even for the perovskites with extensive experimental and computational thermodynamic data (Section 3.1). More effective and efficient schemes using computational screening and ML approaches to predict and select promising candidates from large datasets are thus needed (Section 6). More to the points, the interactions between point defects such as dopant-vacancy and dopant-proton associations are often treated separately in computation due to their complexity. However, as they involve the same type of defects, these defect interactions should be correlated with each other and are expected to influence the hydration and proton diffusion in a non-independent manner. Identifying physical quantities that govern such correlations would also be worthy of further consideration, as it aids in the interpretation and exploration of proton-conducting oxides (Section 7).

One of the reasons of these challenges still persist today is the explosive increase in computational cost associated with the large number of point defect configurations. However, the growth of supercomputers and ML techniques is rapidly resolving this issue. For example, thousands of energy calculations for point defects can now be carried out in a week or a month, by fully utilizing modern supercomputing systems [26–28]. The application of regression models, such as random-forest models, to the large amount of computational data makes it possible to understand the general trends of the property of interest, and to find the dominant factors of it [6,29,30]. For a certain host-dopant combination, ML models or ML interatomic potentials trained by *ab initio* computational data can predict the energy of a realistic configuration of point defects in a few seconds within a reasonable error, and can perform long MD simulations spanning several nanoseconds within a realistic time [5,31]. Therefore, it may be not the speed of computation that currently limits material research, but the development of new computational framework. Incorporating high-performance computing and ML techniques into the study for more complex and realistic point defect configurations will pave the way for new, deep, and general insights into the role of point defects in governing proton conduction in oxides (Sections 6-10).

In this review, we first summarize the efforts that have been made so far to synthesize, evaluate, and elucidate the point defect behavior of proton-conducting oxides both experimentally and computationally. Then, our new computational methodologies are introduced, which have been developed to resolve the issues regarding complex defect interactions discussed above. ML techniques, which are useful for understanding and leveraging the computational and experimental results, are also given.

Section 2 introduces the basic theory of point defects, which is necessary to understand the phenomena of proton conduction in inorganic solid oxides. Section 3 covers experimental methods for identifying the thermodynamics parameters of hydration reaction and the results accumulated for perovskite oxides. Section 4 briefly introduces experimental knowledge of proton diffusion. Section 5 summarizes the representative results of calculations that have been done so far, namely defect formation energy and proton diffusivity. Section 6 introduces our recent work regarding materials discovery with experimental database for perovskites and high-throughput computation for non-perovskites with the help of ML. Section 7 provides new concepts, namely oxygen and hydrogen affinities, that can correlate hydration and proton diffusion and thus are suitable for evaluating the performance of proton-conducting oxides in a computational way. Section 8 shows the results of using *ab initio* calculations and ML techniques to elucidate how the interactions between dopants, protons, and lattice affect proton diffusion. Section 9 explains ML potentials trained by *ab initio* calculations and shows its application to long-time simulations of proton diffusion covering intermediate temperature range. Section 10 introduces replica exchange Metropolis Monte Carlo methods to reveal the realistic defect configurations at a finite temperature, which play a critical role in determining proton conduction. All of these have solved some of the long-standing issues of proton-conducting oxides. Finally, we will present conclusions and perspectives in Sections 11 and 12, the latter shows the direction of future research, combining the developed methodologies and experiments. Through this review, we hope to show that it is now possible to deepen our fundamental understanding of complex defect interactions in proton-conducting oxides and accelerate materials discovery, by taking full advantage of supercomputers and the developed computational/ML methodologies.

## 2. Defect chemistry in proton-conducting oxides

**Figure 1.**
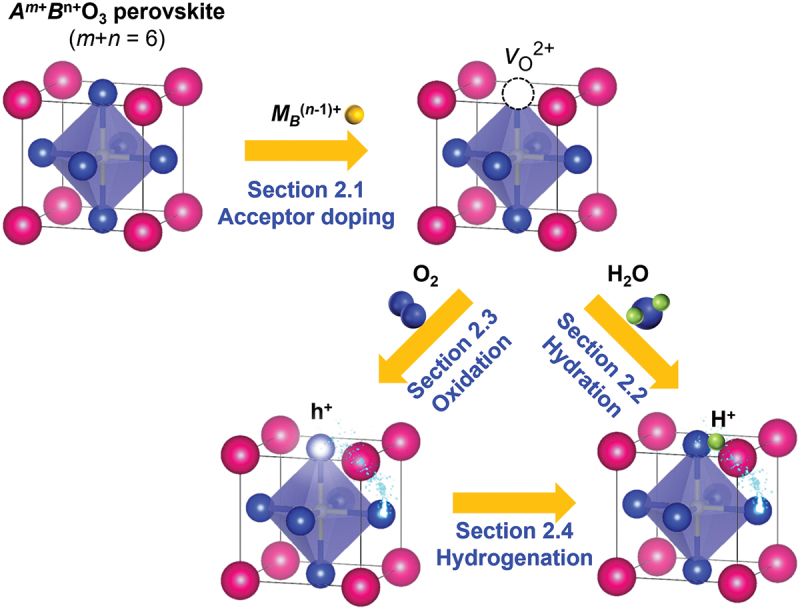
Defect chemistry for activating proton conduction in perovskite oxides. The numbers shown above the reaction indicate the chapter numbers for the respective reactions.

Proton conduction in oxides can be activated by incorporating hydroxyl groups, where the proton forms a covalent bond with the oxide ions. This activation process involves two steps: acceptor doping and hydration reaction. Figure 1 demonstrates this process for perovskite oxides. In the first step, the partial replacement of host cations with lower valence cations results in the formation of oxygen vacancies to maintain the charge balance. In the subsequent hydration reaction, protons are incorporated into the oxygen vacancies by reacting with water molecules in the gas phase. Therefore, the development of proton-conducting oxides requires careful selection of a suitable combination of host-dopant.

Although the scheme may seem simple, the thermodynamics of defect reactions in oxides are complex. There are three main requirements for activating proton conduction in oxides. First, the solution energies of acceptor dopant species in a host compound must be low enough for the acceptor to occupy the host site. Secondly, the doped system must be satisfactory in oxygen affinity, allowing the created oxygen vacancies to be filled by oxygen. Finally, it is crucial that oxidation reactions, which introduce oxygen and electronic holes, are not favored, but rather, hydration reactions should occur. The thermodynamic parameters of these reactions determine the concentrations of hydroxyl groups, oxygen, electronic holes, and oxygen vacancies. These concentrations are influenced by temperature, oxygen and water partial pressures, and the combination of host and dopant. This chapter explains the defect chemistry of proton incorporation and its fundamental thermodynamics.

### 2.1. Acceptor doping

Acceptor doping in host oxides creates oxygen vacancies [14,32]. This reaction for a simple metal oxide, AOn/2, can be expressed in Kröger-Vink notation [33] as

(1) 2MO(n−1)/2+2AA×+OO×→2MA′+vO∙∙+2AOn/2.

Here, A and M are the host and dopant cations that possess the valence of n and n−1, respectively, and vO∙∙ is the oxygen vacancy. The solution energy of an acceptor dopant for an oxide, ΔGsol, can be written as

(2) $$\Delta G\mathrm{sol}=\Delta Gsol\circ +\mathrm{RT}\ln\frac{aMA\prime 2avO∙∙aAOn/22}{aAA\times 2aOO\times 2aMO(n-1)/22},$$

where ai represents the activity of i, and ΔGsol∘ is a Gibbs free energy change for acceptor doping at the standard state,  ∘ [34]. Assuming an ideal solution where there is no interaction between point defects, the equilibrium constant of the solution of acceptor dopant in the oxide is expressed as

(3) $$K\mathrm{sol}=\frac{[MA\prime ]2[vO∙∙]\left[AOn/2\right]2}{[AA\times ]2[OO\times ]2\left[MO(n-1)/2\right]2}=\exp(-\frac{\Delta Gsol\circ }{\mathrm{RT}})=\exp(\frac{\Delta Ssol\circ }{R})\exp(-\frac{\Delta Hsol\circ }{\mathrm{RT}}),$$

where brackets [ ] denote the concentration of each defect and oxides [34], R is the gas constant, T is the absolute temperature, whereas ΔSsol∘ and ΔHsol∘ represent the standard entropy and enthalpy change for acceptor solution, respectively.

The solution energy of an acceptor dopant for an oxide is defined as the energy required to proceed with the reaction of Eq (1). It essentially denotes the difference in free energy between the host oxide in a defect-free state and those containing dopant and oxygen vacancy. The chemical potentials of MO(n−1)/2 and AOn/2 in Eq (1) also affect the solution energy. To determine the solution energy in a complex oxide that contains more than two cations as the host phase, one must consider the chemical potentials of coexisting phases (more details are provided in Section 6.3).

### 2.2. Hydration reaction

Hydration is essential for activating proton conduction in oxides. When oxides come into contact with moisture, oxygen vacancies can be replaced with hydroxyl groups,

(4) $$vO∙∙+OO\times +H2O(g)\overset{K\mathrm{hyd}}{\to }2OHO∙.$$

The equilibrium constant for this hydration reaction is represented as Khyd. In the ideal solution limit where defects do not interact, the equilibrium constant for Eq (4) can be expressed as follows:

(5) $$K\mathrm{hyd}=\frac{[OHO∙]2}{[vO∙∙][OO\times ]pH2O}=\exp(\frac{\Delta Shyd\circ }{R})\exp(-\frac{\Delta Hhyd\circ }{\mathrm{RT}}).$$

Here, pH2O, R, T, ΔShyd∘, and ΔHhyd∘ represent the partial pressure of water, gas constant, absolute temperature, the standard hydration entropy, and the standard hydration enthalpy, respectively. Eq (4) shows that the theoretical maximum of proton concentration is equal to twice the oxygen vacancy concentration, which is determined by the acceptor concentration as shown in Eq (1). The materials parameters ΔShyd∘ and ΔHhyd∘ and the ambient conditions T and pH2O in Eq (5) determine the proton concentration. This fundamental relationship of hydration played an important role in identifying an unknown proton-conducting oxide, which will be explained in Section 6.2.

### 2.3. Oxidation reaction

The oxidation reaction competes with hydration in an oxidizing atmosphere. When oxygen vacancies are filled with oxide ions by the oxidation reaction, electronic holes, h∙, would be introduced in the solids,

(6) $$vO∙∙+\frac{1}{2}O2\left(g\right)\overset{K\mathrm{ox}}{\to }OO\times +2h∙.$$

Here, the location of h∙ is not specified and is considered as free holes in the system. The equilibrium constant of oxidation, Kox, can be denoted as

(7) $$K\mathrm{ox}=\frac{[OO\times ][h∙]2}{[vO∙∙]pO21/2}=\exp(\frac{\Delta Sox\circ }{R})\exp(-\frac{\Delta Hox\circ }{\mathrm{RT}}),$$

where ΔSox∘ and ΔHox∘ represent the standard entropy and enthalpy of oxidation, respectively. A larger value of Kox indicates a stronger driving force for oxide ions to occupy oxygen vacancies, which is basically a measure of ‘oxygen affinity’. Recently, we found that the oxygen affinity also determines the proton conductivity as well as hydration behavior of oxides. This will be explained in Section 7.

### 2.4. Hydrogenation reaction

Hydrogenation reaction is another route to produce protonic carriers by consuming holes,

(8) $$2OO\times +2h∙+H2O\overset{K\mathrm{hydrog}}{\to }2OHO∙+\frac{1}{2}O2.$$

The equilibrium constant of hydrogenation is represented as

(9) $$K\mathrm{hydrog}=\frac{[OHO∙]2pO21/2}{[h∙]2[OO\times ]2pH2O}=\exp(\frac{\Delta Sh\mathrm{ydrog}\circ }{R})\exp(-\frac{\Delta Hh\mathrm{ydrog}\circ }{\mathrm{RT}}),$$

where ΔShydrog∘ and ΔHhydrog∘ represent the standard entropy and enthalpy of hydrogenation, respectively. The degree of hyrogenation is linked to those of hydration and oxidation reactions via Khydrog=Khyd/Kox. Therefore, the hydrogenation reaction indicates which defect would be more stable in the system: either a proton or an electronic hole.

## 3. Experimental determination of hydration thermodynamics of oxides

In this section, we will explain the experimental methodologies used to determine hydration thermodynamics in oxides, present the results obtained from these experiments, and discuss the factors that influence the hydration reaction.

### 3.1. Phenomenological methodology to determine hydration thermodynamics of oxides by experiments

Thermogravimetry is a common experimental method used in this field to determine proton concentration and its hydration thermodynamics such as ΔHhyd∘ and ΔShyd∘. This technique measures the increase in weight that occurs when water molecules from the gas phase are incorporated into the solid. The proton concentration is a function of temperature and water partial pressure as shown in Eq (5).

**Figure 2.**
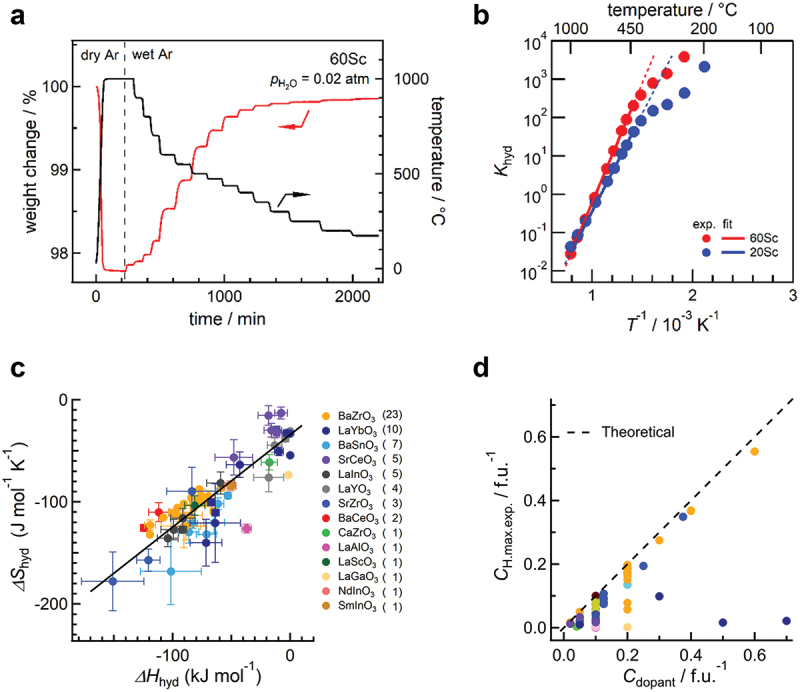
Example of (a) thermogravimetry experiments using BaZr0.4Sc0.6O3−δ, (b) van’t Hoff plot for hydration reaction, (c) relationship between hydration enthalpy and entropy, and (d) plot of maximum proton concentration determined at a temperature vs dopant content. The dashed line in (d) shows the theoretical maximum of proton concentration. The data in (a) and (b) are from [24], whereas one in (c) and (d) are from [35]. The former is under the CC-BY 4.0 license, and the latter is reprinted with permission from American Chemical Society. Copyright 2021 American Chemical Society.

Figure 2a shows an example of a thermogravimetry measurement conducted on BaZr0.4Sc0.6O3−δ [24]. Initial dry heating up to 1000  ∘C dehydrates the sample, which establishes the reference point of zero proton concentration in the oxide. After several hours of dehydration, the atmosphere was changed to a humidified atmosphere with a specific water partial pressure. The introduction of a protonic defect into the oxide would result in weight gain due to water uptake. Here, it is assumed that hydration is the only reaction causing the change in weight. However, it is important to consider that other reactions, such as oxidation in Eq (6), could also contribute to weight change. Therefore, it is crucial to be cautious when performing experiments for each specific condition. By substituting the obtained equilibrium proton concentration into Eq (5), the equilibrium constant of the hydration reaction, Khyd, at a given temperature can be determined. Plotting Khyd against the inverse of absolute temperature produces the van’t Hoff plot (Figure 2b). Furthermore, by finding a linear relationship between ln(Khyd) and T−1 in Eq (5), the values of ΔHhyd∘ and ΔShyd∘ can be determined.

Static calorimetry combined with thermogravimetry is another technique to determine the absolute hydration enthalpy and proton concentration. This method involves measuring the heat flow during hydration (hydration enthalpy) using static calorimetry. Simultaneously, the proton concentration can be measured using thermogravimetry, wherein the atmosphere is changed between dry and wet at a constant temperature [36]. Previous studies have shown that the hydration enthalpy values obtained from both calorimetry and thermogravimetry align well [36–39], highlighting the reliability of thermogravimetry in determining hydration thermodynamics.

#### 3.1.1. Hydration thermodynamics in perovskite oxides

Hydration thermodynamics of proton-conducting oxides within the perovskite category are summarized in Table 1. The host compounds includes BaZrO3 [24,40–43,53], BaCeO3 [43–45], BaSnO3 [43,47,48], BaTiO3 [38,43], BaFeO3 [49], Ba(Nb2/3Ca1/3)O3 [43], SrCeO3 [43,54,55], SrZrO3 [43,55], SrTiO3 [43], SrHfO3 [43], SrFeO3 [49], LaYbO3 [51,52], LaScO3 [52], LaYO3 [52], LaInO3 [52], LaAlO3 [52], and the solid solution system of Ba(Ce,Zr)O3 [44], Sr(Ce,Zr)O3 [55], La(Yb,In)O3 [52,56], La(Yb,Y)O3 [52], (La,Sr)FeO3 [49], (Ba,Sr)FeO3 [49], Ba(Co,Fe,Zr)O3 [57], (Ba,Sr)(Co,Fe)O3 [49]. Hydration enthalpies and entropies strongly depend on the host-dopant combinations. Specifically, ΔHhyd∘ ranges from −22 to −172 kJmol −1 while ΔShyd∘ ranges from −39 to −166.7 Jmol −1K −1. Section 3.1.2 to Section 3.1.4 explain the trends and descriptors for hydration thermodynamics reported in the literature.

**Table 1.** Hydration enthalpy and entropy determined by thermogravimetry analysis. This table is also provided as supplementary material.

Chemical composition	host	dopant	*C*_dopant_	*T*_sinter_/°C	*∆H*°_hyd_ /kJmol^−1^	*∆S*°_hyd_Jmol^−1^K^−1^	Reference
BaZr_0.4_Sc_0.6_O_3_	BaZrO_3_	Sc^3+^	0.6	1600	−121 ± 2	−117 ± 2	[24]
BaZr_0.8_Sc_0.2_O_3_	BaZrO_3_	Sc^3+^	0.2	1600	−104 ± 1	−96 ± 1	[24]
BaZr_0.8_Y_0.2_O_3_	BaZrO_3_	Y^3+^	0.2	1200	−22 ± 1	−39 ± 1	[40]
BaZr_0.7_Y_0.3_O_3_	BaZrO_3_	Y^3+^	0.3	1200	−26 ± 1	−44 ± 1	[40]
BaZr_0.6_Y_0.4_O_3_	BaZrO_3_	Y^3+^	0.4	1200	−26 ± 1	−41 ± 1	[40]
BaZr_0.9_Sc_0.1_O_3_	BaZrO_3_	Sc^3+^	0.1	1700	−119.4	−124.9	[41]
BaZr_0.9_Y_0.1_O_3_	BaZrO_3_	Y^3+^	0.1	1700	−79.5	−88.9	[41]
BaZr_0.9_Gd_0.1_O_3_	BaZrO_3_	Gd^3+^	0.1	1650	−66.1	−85.9	[41]
BaZr_0.9_In_0.1_O_3_	BaZrO_3_	In^3+^	0.1	1700	−66.6	−90.2	[41]
BaZr_0.98_Y_0.02_O_3_	BaZrO_3_	Y^3+^	0.1	1700	−80.9	−94.4	[41]
BaZr_0.95_Y_0.05_O_3_	BaZrO_3_	Y^3+^	0.05	1700	−79.5	−93.5	[41]
BaZr_0.85_Y_0.15_O_3_	BaZrO_3_	Y^3+^	0.15	1700	−83.4	−92.1	[41]
BaZr_0.8_Y_0.2_O_3_	BaZrO_3_	Y^3+^	0.2	1700	−93.3	−103.2	[41]
BaZr_0.9_Y_0.1_YO_3_	BaZrO_3_	Y^3+^	0.1	1715	−74.3	−86.8	[42]
BaZr_0.9_Y_0.1_O_3_	BaZrO_3_	Y^3+^	0.1	>1700	−75.73	−86.24	[43]
BaZr_0.9_Y_0.1_O_3_	BaZrO_3_	Y^3+^	0.1	1700	−83.3	−91.2	[44]
(Ba_0.97_Y_0.03_)(Ce_0.84_Y_0.06_)O_3_	BaCeO_3_	Y^3+^	0.19	NA	−172	−158	[45]
BaCe_0.98_Y_0.02_	BaCeO_3_	Y^3+^	0.02	NA	−126.6	−126.6	[43]
BaCe_0.9_Y_0.1_O_3_	BaCeO_3_	Y^3+^	0.1	NA	−162.2	−166.7	[43]
BaCe_0.9_Y_0.1_O_3_	BaCeO_3_	Y^3+^	0.1	1700	−123	−113	[44]
BaCe_0.9_Y_0.1_O_3_	BaCeO_3_	Y^3+^	0.1	1400	−122	−119	[46]
BaCe_0.9_Yb_0.1_O_3_	BaCeO_3_	Yb^3-^	0.1	1400	−127	−126	[46]
BaSn_0.5_Y_0.5_O_3_	BaSnO_3_	Y^3+^	0.5	NA	−89.1	−73.7	[43]
BaSn_0.875_Sc_0.125_O_3_	BaSnO_3_	SC^3+^	0.125	1600	−86.36	−120.93	[47]
BaSn_0.875_In_0.125_O_3_	BaSnO_3_	In^3+^	0.125	1600	−69.46	−121.04	[47]
BaSn_0.95_Y_0.05_O_3_	BaSnO_3_	Y^3+^	0.05	1600	−46	−50	[48]
BaSn_0.875_Y_0.125_O_3_	BaSnO_3_	_Y_^3+^	0.125	1600	−59.01	−97.17	[47]
BaSn_0.75_Y_0.25_O_3_	BaSnO_3_	Y^3+^	0.25	1600	−66	−72	[48]
BaSn_0.625_Y_0.375_O_3_	BaSnO_3_	Y^3+^	0.375	1600	−84	−83	[48]
BaSn_0.5_Y_0.5_O_3_	BaSnO_3_	Y^3+^	0.5	1600	−68	−73	[48]
BaSn_0.875_Gd_0.125_O_3_	BaSnO_3_	Gd^3+^	0.125	1600	−46.11	−79.33	[47]
BaCa_0.39_ Nb_0.61_O_3_	BaCa_1/3_Nb_2/3_O_3_	Ca^3+^	0.057	NA	−68.7	−108.1	[43]
BaZr_0.7_Ce_0.2_Y_0.1_O_3_	Ba(Zr,Ce)O_3_	Y^3+^	0.1	1600	−93	−96	[44]
BaCe_0.6_Zr_0.3_Y_0.1_O_3_	Ba(Zr,Ce)O_3_	Y^3+^	0.1	1700	−106	−104	[44]
SrTi_0.98_Sc_0.02_O_3_	SrTiO_3_	Sc^3+^	0.02	1590	−22.9	−99.5	[41]
BaTi_0.3_Sc_0.7_O_3_	BaTiO_3_	Sc^3+^	0.7	1550	−56	−93	[38]
BaTi_0.4_Sc_0.6_O_3_	BaTiO_3_	Sc^3+^	0.6	1550	−53	−102	[38]
BaTi_0.8_Sc_0.2_O_3_	BaTiO_3_	Sc^3+^	0.2	1400	−93	−143	[38]
BaTi_0.3_In_0.7_O_3_	BaTiO_3_	In^3+^	0.7	1400	−68	−125	[38]
BaTi_0.5_In_0.5_O_3_	BaTiO_3_	In^3+^	0.5	1400	−57	−132	[38]
BaFe_0.8_Y_0.2_O_3_	BaFeO_3_	Fe^3+^,Y^3+^	-	1300	−71 ± 2	−142 ± 3	[49]
BaCo_0.8_Y_0.2_O_3_	BaCoO_3_	Co^3+^,Y^3+^	-	1250	−50 ± 3	−133 ± 4	[49]
Ba_0.5_Sr_0.5_FeO_3_	(Ba,Sr)FeO_3_	Fe^3+^	-	1200	−61 ± 3	−150 ± 6	[49]
Ba_0.5_Sr_0.5_Fe_0.8_Zn_0.2_O_3_	(Ba,Sr)FeO_3_	Fe^3+^,Zn^2+^	-	1200	−76 ± 4	−137 ± 5	[49]
BaCo_0.4_Fe_0.4_Zr_0.1_Y_0.1_O_3_	Ba(Co,Fe,Zr)O_3_	Co^3+^,Fe^3+^,Y^3+^	-	1200	−62 ± 2	−149 ± 2	[49]
BaCo_0.4_Fe_0.4_Zr_0.2_O_3_	Ba(Co,Fe,Zr)O_3_	Co^3+^,Fe^3+^	-	1200	−56 ± 7	−137 ± 12	[49]
Ba_0.5_Sr_0.5_Co_0.8_Fe0.2	(Ba,Sr)(Co,Fe)O_3_	Co^3+^,Fe^3+^	-	1200	−50 ± 4	−151 ± 6	[49]
SrFe_0.8_Zn_0.2_O_3_	SrFeO_3_	Zn^2+^	0.2	1200	−30 ± 3	−112 ± 4	[49]
Ba_0.95_La_0.05_FeO_3_	(La,Ba)FeO_3_	Fe^3+^	-	1200	−62 ± 3	−144 ± 5	[49]
Sr_0.75_La_0.25_FeO_3_	(La,Sr)FeO_3_	Fe^3+^	-	1200	−37 ± 2	−143 ± 4	[49]
Sr_0.85_La_0.15_FeO_3_	(La,Sr)FeO_3_	Fe^3+^	-	1250	−39 ± 3	−151 ± 5	[49]
Ba_0.85_La_0.15_FeO_3_	(La,Ba)FeO_3_	Fe^3+^	-	1200	−60 ± 2	−160 ± 2	[49]
Ba_0.75_La_0.25_FeO_3_	(La,Ba)FeO_3_	Fe^3+^	-	1200	−44 ± 4	−143 ± 7	[49]
Ba_0.95_La_0.05_Fe_0.95_Zn_0.05_O_3_	(La,Ba)FeO_3_	Fe^3+^,Zn^2+^	-	1200	−47 ± 5	−106 ± 8	[49]
Ba_0.95_La_0.05_Fe_0.9_Zn_0.1_O_3_	(La,Ba)FeO_3_	Fe^3+^,Zn^2+^	-	1200	−53 ± 6	−109 ± 10	[49]
Ba_0.95_La_0.05_Fe_0.8_Zn_0.2_O_3_	(La,Ba)FeO_3_	Fe^3+^,Zn^2+^	-	1200	−86 ± 5	−143 ± 6	[49]
Ba_0.95_La_0.05_Fe_0.8_Zn_0.2_O_3_	(La,Ba)FeO_3_	Fe^3+^,Zn^2+^	-	1200	−61 ± 4	−150 ± 6	[49]
La_0.9_Sr_0.1_AlO_3_	LaAlO_3_	Sr^2+^	0.1	1700	−66 ± 24	−91 ± 21	[50]
La_0.9_Sr_0.1_ScO_3_	LaScO_3_	Sr^2+^	0.1	1700	−105 ± 9	−116 ± 9	[50]
La_0.9_Sr_0.1_ScO_3_	LaScO_3_	Sr^2+^	0.1	1750	−97 ± 5	−112 ± 5	[24]
La_0.9_Sr_0.1_YO_3_	LaYO_3_	Sr^2+^	0.1	1700	−96 ± 9	−70 ± 6	[50]
La_0.9_Sr_0.1_InO_3_	LaInO_3_	Sr^2+^	0.1	1500	−96 ± 3	−120 ± 6	[50]
La_0.9_Ba_0.1_YbO_3_	LaYbO_3_	Ba^2+^	0.1	1700	−141	−111	[51]
La_0.9_Sr_0.1_YbO_3_	LaYbO_3_	Sr^2+^	0.1	1700	−164 ± 26	−135 ± 21	[50]
La_0.9_Sr_0.1_Yb_0.8_In_0.2_O_3_	La(Yb,In)O_3_	Sr^2+^	0.1	1500 or 1700	−125.5 ± 0.4	−117.2 ± 0.4	[52]
La_0.9_Sr_0.1_Yb_0.5_In_0.5_O_3_	La(Yb,In)O_3_	Sr^2+^	0.1	1500 or 1700	−106.9 ± 1.0	−116.5 ± 1.1	[52]
La_0.9_Sr_0.1_Yb_0.2_In_0.8_O_3_	La(Yb,In)O_3_	Sr^2+^	0.1	1500 or 1700	−117.3 ± 0.8	−136.1 ± 1.3	[52]
La_0.9_Sr_0.1_Yb_0.8_Y_0.2_O_3_	La(Yb,Y)O_3_	Sr^2+^	0.1	1500 or 1700	−131 ± 5.9	−100.2 ± 6.9	[52]
La_0.9_Sr_0.1_Yb_0.5_Y_0.5_O_3_	La(Yb,Y)O_3_	Sr^2+^	0.1	1500 or 1700	−93.6 ± 17.4	−65.5 ± 20.1	[52]
La_0.9_Sr_0.1_Yb_0.2_Y_0.8_O_3_	La(Yb,Y)O_3_	Sr^2+^	0.1	1500 or 1700	−141.7 ± 10.0	−102.3 ± 11.6	[52]

#### ΔShyd∘vs ΔHhyd∘

3.1.2.

A correlation has been proposed by Kreuer between ΔShyd∘ and ΔHhyd∘, where a more negative ΔHhyd∘ leads to a more negative ΔShyd∘ [14]. This relationship was reported using the hydration thermodynamics for BaCeO3, BaZrO3, BaCa1/3Nb2/3O3, BaSnO3, and SrTiO3-based oxides [14]. The relationship has been held against a larger data set of 65 perovskite oxides, as shown in Figure 2c. Although the underlying physical chemistry of this relationship is not fully understood, such a relationship is generally found as entropy-enthalpy compensation for all reactions [58] (pre-exponential factor-activation energy relationships for conductivity, the Meyer–Neldel rule [59], for atomic diffusion in metals [60], minerals [61], semiconductors [62], and ionic crystals [63]).

#### CHvs Cdopant

3.1.3.

Heavy acceptor doping increases the concentration of protons in oxides (Figure 2d). The experimental results indicate that the proton concentration generally rises as the amount of acceptor dopant increases. It is important to note that the proton concentration cannot exceed the theoretical maximum defined by the number of oxygen vacancies, as hydration follows the mechanism expressed in Eq (4). This theoretical maximum is represented by the dashed line in Figure 2d, and it is observed that most oxides adhere to this correlation. This simple correlation has been deemed essential for predicting proton concentration and for the discovery of new proton-conducting oxides through ML. These findings will be further elaborated on in Section 6.

#### 3.1.4. Traditional descriptors for hydration of oxides

##### 3.1.4.1. Basicity of oxide ions

The basicity of oxide ions is proposed as a factor that influences on hydration enthalpy of perovskite oxides [14,41,43,45]. Since the basicity is a general concept describing how strong a base is, no study has reported the value of basicity for proton-conducting oxides. It is proposed that the Brønsted basicity of oxide ions would be expressed by the electronegativity of cations bonded to oxide ions [14]. Smaller electronegativity (higher acidity) of O-bonded cations (*i.e*. B-site for perovskite structure) may increase electron density at the oxide ions (higher basicity) and strengthen the covalent bond of O-H toward more negative hydration enthalpy. When the Pauling electronegativities [64] of the B-site cation, shown in parentheses, become small, the hydration enthalpy tends to show more negative values from titanates (1.54) to niobates (1.6), stannates (1.96), zirconates (1.33), and cerates (1.12).

Other expressions for the oxygen basicity in perovskites are the difference of weighted average in electronegativity of A-site cations from that of B-site ones, ΔχB−A [15] and the weighted average of Zi/ri2 (the ion electronegativity, where Zi is the charge of cation and ri is the Shannon ionic radii [65] for cation species i) [57]. The former was proposed based on hydration data for 40 compositions (the chemical compositions were not given) [15] whereas the latter was based on the standard Gibbs free energy change upon hydration, ΔGhyd∘, at 700 K for 13 compositions (11 host of BaPrO3, BaCeO3, BaZrO3, LaErO3, LaYbO3, SrZrO3, CaZrO3, BaTiO3, SrTiO3, LaScO3, and BaSnO3) [57]. Decreases in both parameters, ΔχB−A and Zi/ri2, show a tendency of more negative ΔHhyd∘ and ΔGhyd∘ at 700 K.

##### 3.1.4.2. Molar density of oxides

Larring and Norby proposed that hydration enthalpies could correlate with the molar density of oxides [66]. As the molar density of the oxide increases (typically associated with heavier elements and reduced lattice volumes), the hydration enthalpy becomes increasingly negative. This is supported by hydration behavior observations in perovskite oxides (Yb-doped SrCeO3, Nd-doped BaCeO3, Ca-doped LaErO3) and rare-earth oxides (Ca-doped La2O3, Gd2O3, Dy2O3, Y2O3, Er2O3, LaGdO3, and GdErO3).

##### 3.1.4.3. Structural symmetry

Symmetry in the crystal structure has been proposed as a factor that influences proton concentration in perovskite oxides. While the maximum proton concentration ideally increases with the dopant concentration, reducing crystal symmetry from cubic, *i.e*. BaZrO3, to orthorhombic, *i.e*. SrZrO3, SrCeO3, and BaCeO3, may reduce the saturation limit of protons [14].

The tolerance factor [67], an indicator of structural symmetry in perovskites, serves as a descriptor for the hydration enthalpies of 40 chemical compositions, including BaZrO3, BaCeO3, and PbZrO3. The results indicate that a smaller tolerance factor correlates with more negative hydration enthalpies [36,68].

##### 3.1.4.4. Site selectivity of acceptor dopant

The site selectivity of dopants in perovskite crystals also influences the thermodynamics of hydration reaction. In some cases, acceptors that are intended to replace a specific site end up occupying another site [15,20]. When trivalent acceptor doping occurs at the B-site of 2+/4+ perovskites (M3+-doped A2+B4+O3−δ), partitioning at the A-site results in a reduction of oxygen vacancy concentration [69],

(10) M2O3+2AA×+vO∙∙→2MA∙+OO×+2AO.

Thus, the concentration of protons would be reduced. Lower water uptake has been observed in Ba-deficient Gd-doped BaCeO3 [45,69] and Y-doped BaZrO3 [53,70]. The introduction of cation non-stoichiometry during synthesis and high-temperature sintering significantly alters the hydration behavior of the oxide, despite identical nominal compositions [69].

### 3.2. Local structure probed by experimental methods

The hydration reaction is believed to be influenced by the local environments surrounding the oxygen vacancies in the perovskite structure, as a water molecule occupies the oxygen vacancy site. The oxygen vacancies are located between two B-site cations, either a host B cation or an acceptor cation, M, creating three potential configurations: M−vO∙∙−M, M−vO∙∙−B, and B−vO∙∙−B. These configurations are based on the first nearest neighbor cation sites of the oxygen vacancies. It is well known that oxygen vacancies are associated with acceptor dopants [71,72] due to the electrostatic interaction between the relatively positive vO∙∙ and the negative acceptor cations MB′. This indicates that the stability of the oxygen vacancy depends on the local configuration, which in turn affects the hydration enthalpy.

The changes in local structure around B-site cations due to hydration have been investigated using X-ray absorption spectroscopy (XAS) [73–76] and solid-state nuclear magnetic resonance (NMR) [77–80]. Extended X-ray absorption fine structures (EXAFS) obtained from *ex-situ* XAS have been reported for dry 2–30 at% Y-doped barium cerates [73,74], 12–75 at% In-doped barium zirconates [75], and dehydrated and hydrated 6–15 at% Y-doped barium zirconates [76]. EXAFS at low temperatures of −196 or −248  ∘C has shown local disorder or enlargement around Y dopants compared to the host B-site cations in doped barium cerates and zirconates [74,76]. Solid-state NMR has provided additional insights into the local structures surrounding protons and specific dopants in 5–40 at% Sc-doped barium zirconates [77,78,80], 20 and 30 at% Y-doped barium zirconates [81,82], and Sc-doped barium stannates [79]. For example,  45Sc NMR of dehydrated and hydrated 15 at% Sc-doped barium zirconates at room temperature has shown the presence of oxygen vacancies near Sc dopants (ScO5), which are then filled to form ScO6 after hydration [77]. Despite some indications of changes in the local structure during hydration, the oxygen vacancy environment that is most favorable for hydration in perovskite oxides remains elusive. In Section 10, we explore this aspect using state-of-the art computational methodology employing machine-learning and thermodynamic sampling algorithms in combination with in situ XAS and thermogravimetry.

## 4. Proton diffusion and conduction in oxides

Proton diffusivity is also a crucial factor for determining proton conductivity. The development of fast proton-conducting oxides has been an effort to design materials that possess high proton concentration and diffusivity (mobility). Here, we outline proton diffusion and conduction in oxides in a phenomenological way and summarize the proposed descriptors. Computational studies in proton diffusion will be described in Section 5.

### 4.1. Proton diffusion and trapping in oxides

Proton transport is believed to occur through a Grotthuss-like mechanism, which involves rotation and hopping motions at oxygen sites [83]. After the hydration reaction, protons occupy four sites located approximately 1 Å away from the oxygen sites [84,85]. *Ab initio* MD simulations illustrate that these introduced protons rotate around the oxide ion of BO6 octahedra and hop between the proton sites [19,86]. Experiments such as quasielastic neutron scattering [87–89] and the observation of light-heavy hydrogen isotope effect on conductivity [90,91] also support the Grotthus-like mechanism.

The temperature dependence of proton diffusivity, DH, is often explained by the transition state theory combined with the random walk theory [92],

(11) $$DH=\frac{f\Gamma d2}{6},$$

where f, Γ, and d denote the correlation factor, jump frequency, and jump distance, respectively. The jump frequency, Γ, is given as

(12) Γ=Zpν.

Here, Z is the number of equivalent jump sites, p is the jump probability, and ν is the oscillation frequency. When the change in free energy between stable and transition sites for carriers is given by ΔGm∗, the jump probability is expressed as

(13) $$p=\exp(-\frac{\Delta Gm*}{\mathrm{RT}})=\exp(\frac{\Delta Sm*}{R})\exp(-\frac{\Delta Hm*}{\mathrm{RT}}).$$

Substituting Eq (12) and (13) into Eq (11) results in

(14) $$DH=\frac{fz\nu d2}{6}\exp(\frac{\Delta Sm*}{R})\exp(-\frac{\Delta Hm*}{\mathrm{RT}})=D0\exp(-\frac{Ea}{\mathrm{RT}}),$$

where $D0=\frac{fz\nu d2}{6}\exp(\frac{\Delta Sm*}{R})$ and Ea=ΔHm∗, respectively. This is widely recognized as the Arrhenius equation, which illustrates a linear relationship between the logarithm of proton diffusivity and the inverse of absolute temperature (1/T). To promote proton diffusion, it is vital to obtain a lower migration barrier and higher pre-exponential value. These terms are correlated with the jump distance, jump frequency, and entropy change between stable and transitional states.

Although the Arrhenius equation suggests a linear relationship between the logarithm of DH against 1/T, it is known that proton diffusivity exhibits non-linear behavior. This non-linear macroscopic proton transport is due to proton trapping, experimentally proven in the 20 at% Y-doped barium zirconate by Yamazaki et al. [81]. The simplest expression of mobile and trapped protons, OHO.m∙ and OHO.trap∙, respectively, in local equilibrium is as follows [93]

(15) $$OHO.m∙\overset{K\mathrm{trap}.H}{\to }OHO.\mathrm{trap}∙.$$

This expression does not specify the mechanism of proton trapping.

One possible mechanism for trapping is proton-dopant association. The non-linear behavior has been derived phenomenologically in Ref. [81] assuming this mechanism:

(16) $$OHO.m∙+MB\prime \overset{K\mathrm{as}.H}{\to }(OHO∙MB\prime )\times .$$

The analysis assumes an ideal solution as a first approximation so that deviations from ideal behavior must be considered for materials such as heavily Sc-doped barium zirconates with high proton concentration, where interactions between dopants and protons are anticipated [24]. Additional proposed mechanisms for proton trapping include the B-O-B angle of metal-oxygen octahedra in perovskites [94].

Regardless of the specific mechanisms for proton trapping, trapped protons require additional energy to escape from their sites for macroscopic diffusion [81,93]. This reduces the number of mobile protons at lower temperatures, resulting in a downward curvature in the Arrhenius plot of proton diffusivity.

### 4.2. Proton conduction in oxides

Proton conductivity can be expressed as the product of proton concentration and mobility,

(17) σH=FCHμH,

where F and μH are the Faraday constant and proton mobility, respectively. Based on the Nernst-Einstein relationship, proton mobility relates to proton diffusivity in

(18) $$\mu H=\frac{FDH}{\mathrm{RT}}.$$

Inserting Eq (18) into Eq (17) leads to

(19) $$\sigma H=\frac{F2CHDH}{\mathrm{RT}}.$$

Thus, Arrhenius representation for proton conductivity is expressed as

(20) $$\sigma HT=\frac{F2CHD0}{R}\exp(-\frac{Ea}{\mathrm{RT}}).$$

The apparent activation energy for proton conductivity can be obtained from the slope of the ln(σT) - 1/T plot. Since the carrier concentration in proton-conducting oxides is, unlike the oxide ion conductors like yttria-stabilized zirconia, a function of temperature, the apparent activation energy does not directly reflect that for the proton diffusivity (mobility).

### 4.3. Traditional descriptors for proton diffusion (association) and conduction

Descriptors for proton diffusion and conduction serve as critical guides for materials development. Researchers have proposed such descriptors that correlate the dynamics of protons in oxides with the static characteristics of compounds. These descriptors can be categorized into two distinct approaches: fundamental understanding versus crystallographic features that can be controlled through the selection of chemical compositions and synthesis methods. Chung et al. adopted the former approach, focusing on hydrogen bonding and phonon modes [95]. In this review, we emphasize the latter approach, which aids in the selection of chemical compositions and the synthesis of materials for development. Despite several proposed descriptors for proton diffusion and conduction, the interdependence of these descriptors complicates the representation of proton dynamics based on the static properties of crystals.

#### 4.3.1. Increased lattice volume for weaker hydrogen bonding

The increased lattice volume has been considered beneficial in increasing proton diffusivity and, thus, proton conductivity [14]. This would arise from an enlarged hydrogen bonding angle, which destabilizes hydrogen bond formation and subsequently lowers the activation energy required for proton diffusion.

#### 4.3.2. Structural symmetry

Cubic symmetry has been posited to enhance proton diffusivity [14]. Comparative analysis of cubic BaZrO3 and orthorhombic SrZrO3 shows that the former demonstrates an activation energy for proton conduction of approximately ∼45 kJmol −1 [41,81,96], whereas the latter exhibits a higher value of around ∼60 kJmol −1 [43]. Additionally, the tolerance factor serves as a critical parameter reflecting the structural symmetry of perovskite, potentially correlating with proton diffusivity and conductivity.

#### 4.3.3. Ionic radius of acceptor dopant

The dopant size significantly influences proton conductivities in perovskite compounds [14,97–99]. Shannon ionic radii [65] characterize the ionic radius of the acceptor dopant. In barium zirconate, the selection of Y as a dopant, with an ionic radius of 0.90 Å in six-coordinate environments, exceeding the 0.72 Å of Zr, results in peak proton conductivity for 10–20 at% Y dopant concentration at approximately 100  ∘C [14,97–99]. This phenomenon has been attributed to macroscopic and local structural effects [14] and proton-dopant association [20,81]. Calculations of enthalpies of proton-dopant association in doped barium zirconates indicate that Y dopant exhibits one of the lowest association energies [20]. Reduced association energy would correlate with lower apparent activation energy, thereby enhancing proton diffusivity [81].

#### 4.3.4. Dopant concentration

**Figure 3.**
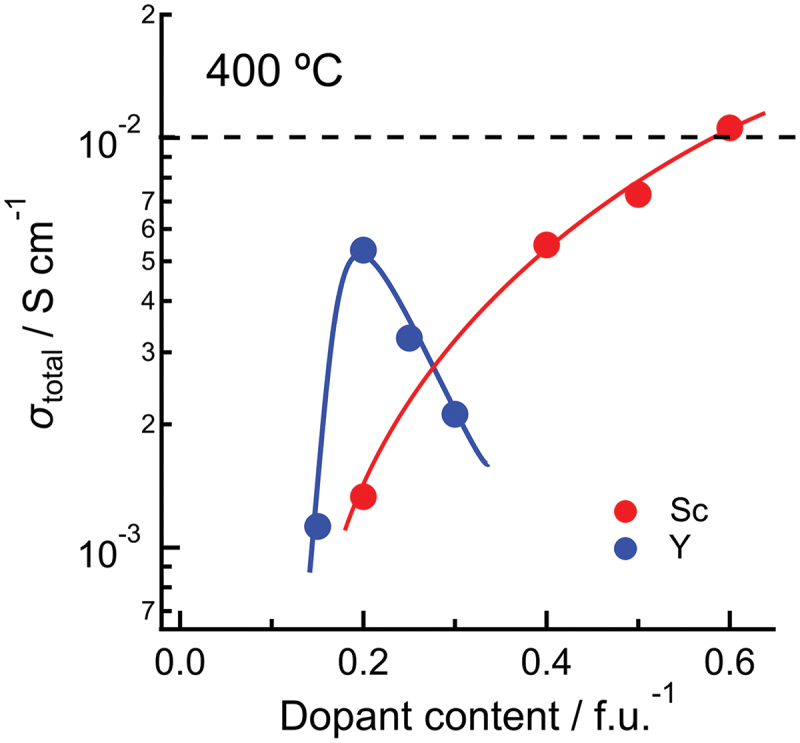
Proton conductivity at 400 ∘C against dopant concentration. A part of the data for Sc-doped barium zirconate is referred from [24] the data for Y-doped barium zirconate are from [100].

The choice of dopant-host combination and dopant concentration significantly impact the proton conductivity. When using barium zirconate as the host, the highest proton conductivity had been reported for Y dopant at 20 at% below its solubility limit with further Y doping leading to a decrease in the conductivity [70,100–102]. This implies that the mobility of protons decreases with increasing dopant concentration. Recently, however, some of the authors reported the opposite concentration dependence for Sc doping, *i.e.* continuous conductivity increase up to the solubility limit of 60 at% [24] as shown in Figure 3. Attempts have been made to understand the influence of the dopant type and concentration on the conductivity, *e.g.* in terms of proton trapping by isolated dopants [81]. However, a clear picture is yet to emerge especially for high dopant concentration, where a simple two-state picture of trapped and mobile protons (Eq 15–16) breaks down because dopants are no longer dilute and mutually isolated. Recent computational works including ours have shed some light on this issue and are reviewed in Sections 5–10.

## 5. Computation of thermodynamics and dynamics of defects in proton-conducting perovskite oxides

In this section, we summarize previous computational efforts that have been performed for understanding the microscopic behavior of defects in proton-conducting perovskite oxides. The calculations for proton-conducting oxides can be divided into static energy calculations and dynamic diffusion calculations. The former analyzes the interactions of dopants, oxygen vacancies, and protons that determine the concentrations and configurations of defects, while the latter investigates the diffusion of protons in a given defect (dopant) configurations. These two kind of calculations are described in Section 5.1 and 5.2, respectively.

### 5.1. Static calculation for defect formation

As noted in Section 3, hydration behavior depends not only on the dopant content, but also on the dopant species and processing conditions. This means that although the defect chemistry of acceptor doping and hydration can be understood from charge neutrality arguments, dopant species behave quite differently at the nanoscale due to the different interactions with oxygen vacancy, proton, and host lattice. The processing dependence also suggests that there is a temperature dependence in the dopant arrangements. Probing such nanoscale behavior using experiments is challenging, and many workers have resorted to *ab initio* simulations for understanding defect formation behavior.

A way typical to obtain insights into defect chemistry from *ab initio* calculations is to start from defect formation energy calculations. A defect formation energy is defined as the excess energy necessary for creating a point defect in a perfect crystal system [103] and can be calculated by the following equation [104,105],

(21) $$\Delta G\mathrm{def}(Dq)=G\mathrm{def}(Dq)+E\mathrm{corr}(Dq)-G\mathrm{perf}+\sum_{\begin{array}{c} X \end{array}}\Delta nX\mu X+q(E\mathrm{VBM}+\Delta EF),$$

where Gdef(Dq) is the free energy of the system with a single point defect Dq, Gperf is that of a perfect reference system without any defects, μX is the chemical potential of species X related to the point defect, ΔnX is the difference in number of species X between the defective and the perfect systems with a plus sign for a vacancy and a minus sign for an interstitial and an impurity, q is the charge state of the defect, EVBM is the valence band maximum (VBM) energy, and ΔEF is the Fermi level position measured from the VBM. Ecorr(Dq) is an energy correction term for a supercell calculation including a charged defect under the periodic boundary condition [105,106]. It should be noted that ΔGdef(Dq) corresponds to an excess energy to form a single point defect Dq and does not include configuration entropy contributions. The chemical potential μX can be calculated from the thermal equilibrium condition of coexisting phases. For example, when a compound MXn is equilibrated with a simple substance of M, the chemical potentials of μM and μX are determined as

(22) $$\left\{\begin{array}{c} \mu M=G(M)\\ \mu X=\frac{G(MXn)-G(M)}{n} \end{array}\right.$$

where G(M) and G(MXn) are the free energies of compounds M and MXn per formula unit, respectively. Therefore, the value of the chemical potentials of μM and μX can be evaluated from calculating the energy of the coexisting phases, M and MX. If X is a species of a gas phase, μX can be related to the partial pressure and temperature. Considering oxygen defects as a concrete example, a chemical potential of oxygen at a temperature T and a partial pressure pO2, μO(T,pO2), can be expressed as

(23) $$\begin{array}{cc} \mu O(T,pO2) & =\frac{1}{2}\mu O2(T,pO2)\\ & =\frac{1}{2}\{\mu O2\circ (T,p\circ )+\mathrm{kT}\ln(pO2/p\circ )\}\\ & =\frac{1}{2}\{HO2(0K,p\circ )+\Delta HO2(T,p\circ )-TSO2\text{\textbackslash{}break}\;\;\;\;\;\;\;(T,p\circ )+\mathrm{kT}\ln(pO2/p\circ )\}, \end{array}$$

where μO2∘(T,p∘) is the chemical potential of O2 gas at an absolute temperature T and a standard pressure p∘, HO2(0K,p∘) is an enthalpy of O2 at 0 K and p∘, ΔHO2(T,p∘) is a difference of enthalpies of O2 between a given T and 0 K, SO2(T,p∘) is entropy of O2 at T and p∘, and k is the Boltzmann constant. The values of ΔHO2(T,p∘) and SO2(T,p∘) can be referred from the NIST-JANAF thermochemical tables [107]. If total energies obtained by *ab initio* calculations are assumed to be enthalpies at 0 K, we can determine the chemical potential of a gas phase at the desired temperature and pressure from the combination of *ab initio* calculations and the thermochemical data. From these concepts, the effect of partial pressure and temperature of the atmospheric gas phase can be indirectly involved in the calculations of defect formation energies. It should be noted that the formalism mentioned above does not take into account the influence of the vibration of solid phases on defect formation behavior. If one directly includes vibrational effects, phonon calculations are additionally needed [21,108].

As noted above, ΔGdef(Dq) is the formation energy of single point defect. In the free energy change of the entire system, the configurational entropy due to the formation of a certain concentration of point defects should be taken into account. As an example, we consider formation of nv oxide ion vacancies in an oxide system. If interactions between defects are small enough, the Gibbs free energy change from a perfect state to a defective state can be approximated as

(24) ΔG=nvΔGdef(vO∙∙)−TΔSc

where ΔSc is the configurational entropy. The Boltzmann’s entropy formula gives the relationship,

(25) ΔSc=klnW,

where W is a number of distinct ways in which defects can be arranged on regular sites. If the number of the regular oxygen sites of the oxide is NO, the number of distinct ways of *n* oxide ion vacancies put on the oxygen sites is

(26) $$W=\frac{NO!}{(NO-nv)!nv!}$$

The configurational entropy is rewritten as

(27) $$\Delta Sc=k\ln\frac{NO!}{(NO-nv)!nv!}$$

For large numbers of N and n like mole of atoms, Stirling’s approximation, lnN!=NlnN−N, can be used. Finally, the total Gibbs free energy change is given by

(28) ΔG=nvΔGdef(vO∙∙)−kT[NOlnNO−(NO−nv)ln(NO−nv)−nvlnnv]

Under a thermal equilibrium condition, ΔG shows minima against a variation of nv. This situation corresponds to ∂ΔG/∂nv=0. From this condition, the thermal equilibrium concentration of the oxide ion vacancy is given by

(29) $$\frac{nv}{NO-nv}=\frac{\left[vO∙∙\right]}{\left[OO\times \right]}=\exp\left(-\frac{\Delta G\mathrm{def}(vO∙∙)}{\mathrm{kT}}\right).$$

Once the value of ΔGdef(Dq) is determined, we can evaluate the thermal equilibrium concentration of the defect at a given temperature and a partial pressure [109–115].

Using the above methodologies, previous studies have focused on the oxygen vacancies and proton-dopant associations, typically in acceptor-doped BaZrO3 systems. The association energy is defined as a difference between the energy of a defect cluster and the summation of the energies of isolated defects. For example, the association energy of a pair of an acceptor dopant and a proton is calculated by the following equation,

(30) Gas((MZrOHO)×)=ΔGdef((MZrOHO)×)−{ΔGdef(MZr′)+ΔGdef(OHO∙)}.

Energy calculations have pointed out that the association energies depend on the ionic size of a dopant and the configuration of a dopant-proton pair [20–22,116]. The previous calculations were mainly performed under a dilute condition where calculation models included a single defect or a dopant-proton pair (or a pair of a dopant and an oxide ion vacancy). Many-body defect–defect interactions in actual heavily doped systems have not been realistically taken into account.

Structural descriptors, especially those related to hydrogen bonds, have been used to understand the energies of structures containing dopants and protons [14]. Such structural descriptors should also be helpful in understanding the activation and association energies of proton diffusion. Previous defect calculations showed that local structural distortion such as bent O-H-O angles occurs due to the hydrogen bonding [116]. Systematic calculations for Y-doped BaZrO3 also indicated that stable structures have a specific range of B-OH-B angles [94]. However, these calculations only considered the models with low dopant and proton concentrations, and structural distortions that occurs only in high dopant concentration regions have not been discussed. Thus, it remains challenging to understand, from a structural perspective, the dopant concentration dependence of proton diffusivity that is dependent on dopant species (Figure 3).

### 5.2. Proton dynamics probed by ab initio molecular dynamics and kinetic Monte Carlo simulations

Describing breaking and formation of covalent O-H bonds and hydrogen bonds is virtually impossible using classical interatomic potentials, so simulation of proton diffusion requires computationally demanding calculations based on quantum physics. To this end, many *ab initio* and semiclassical MD simulations of proton diffusion in perovskite oxides have been reported in the literature [19,84,86,94,117–120]. They all report a Grotthus-like mechanism, where protons are bonded to O in the parent lattice and can rotate around and hop between these sites. Moreover, proton diffusivities can be calculated from MD simulation trajectories by first evaluating the mean squared displacement (MSD) as a function of lag time Δt,

(31) MSD(Δt)=⟨|xi(t0+Δt)−xi(t0)|2⟩t0,i,

where xi(t) is the position of the ith proton at time t, and the braket means to take an ensemble average over time origin t0 and proton index i. Then, the self-diffusion coefficient is related to the slope of the MSD at infinite Δt:

(32) $$D=\frac{1}{2n}\underset{\mathrm{\Delta t}\to \infty }{\lim}\frac{d}{d(\mathrm{\Delta t})}\mathrm{MSD}(\Delta t),$$

where n is the dimensionality of the diffusion, which is three in the case of bulk proton conduction in the perovskite oxide.

MD simulations naturally contain local lattice-proton and proton–proton interactions and enables modeling of diffusion beyond random walk theory on a homogeneous lattice; thus, it should be capable of reproducing the non-linearity in the Arrhenius plots discussed in Section 4 and analyzing association effects from a microscopic viewpoint. The challenge is in obtaining long enough trajectories to converge these values; this is relatively easy to achieve when the simulations are performed at higher temperatures where the protons are more mobile. However, *ab initio* MD is often too computationally expensive for achieving convergence at intermediate temperatures where these materials are to be used. Thus, fully reproducing the non-linearity in the Arrhenius plot discussed above from these simulations have not been feasible, and discussion of the association effect from these simulations have been limited to qualitative observations that protons tend to be trapped near acceptor dopants.

An alternative approach for simulating long-time dynamics is the kMC method [121,122], which calculates the dynamics of atomistic systems as a stochastic chain of discrete activated events. At each kMC step, a table of possible elementary processes is generated by considering all possible microscopic events for the atomistic configuration at that step. In the case of protons in perovskite oxide, this consists of rotation and hopping events in various directions for all protons in the supercell. Then, a single event is chosen randomly from the table with a probability proportional to the rate:

(33) $$Pi=\frac{\Gamma i}{\sum k\Gamma k},$$

where i is the index of the chosen event and Γ denotes event rates. When simulating proton diffusion, the necessary diffusion rates can be precalculated for various environments around the proton. For this, a standard approach is to employ the nudged elastic band (NEB) method [123,124] in combination with *ab initio* calculation. The NEB method is an approach for calculating minimum energy paths between locally stable configurations; the energy difference between the saddle point and the energy minima along the minimum energy path corresponds to diffusion barriers ΔEa, which can be related to the microscopic diffusion rate as

(34) $$\Gamma =\nu 0\exp\left(-\frac{\Delta Ea}{\mathrm{kT}}\right),$$

according to harmonic transition state theory [125]. ν0 is calculated from normal modes at the energy minimum and the saddle point as

(35) $$\nu 0=\frac{\prod \;i=1N\nu i0}{\prod \;i=1N-1\nu i}.$$

In practice, however, ν0 is often approximated by a single characteristic frequency since calculation of normal modes in a complex oxide with dopants is computationally expensive. This is often not a bad approximation since the exponential term usually has a much larger impact on the rates.

**Figure 4.**
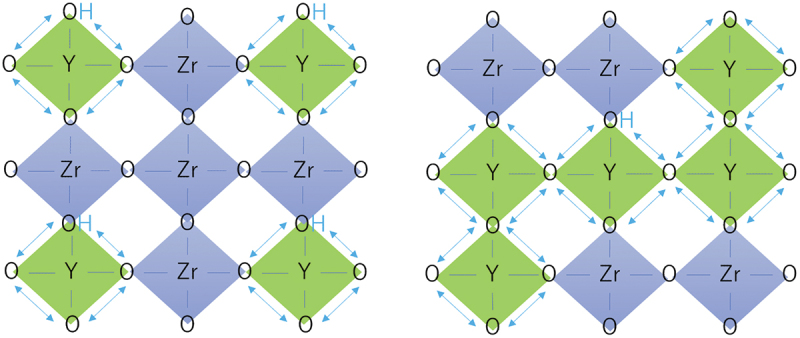
Schematic of proton trapping by isolated dopants (left) and proton diffusion along a long-range network of dopants (right).

With a precalculated table of rates, kMC simulation is typically capable of time propagation of up to micro- or milliseconds, and it has succeeded in reproducing the ‘bending’ in the Arrhenius plot often associated with proton trapping by dopants [23]. kMC simulation has also successfully reproduced the conductivity maximum vs. dopant content in Y-doped BaZrO3 (Figure 3) [23]. From analysis of the proton trajectories, it was found that protons diffuse along a three-dimensional network of dopants, and that the traditional one-to-one dopant-proton trapping/detrapping picture is an oversimplification for more heavily doped systems [23,126]. That is, while protons are confined to (*i.e*. trapped in) regions next to dopants due to the association effect, these ‘trap’ sites are connected to each other and form a long-range migration pathway throughout the crystal (Figure 4). The conductivity maximum, or diffusivity decrease with increasing dopant concentration, was explained by a many-body trapping picture: proton sites surrounded by three or four Y dopants were found to be deeper traps along the three-dimensional migration pathway, and an increase in the number of such configurations was suggested to be the cause for the decrease in diffusivity of the individual protons [23]. On the other hand, proton sites surrounded by Sc doping does not lead to such deeper traps [127], and this explains why Sc doping leads to a continuous conductivity increase up to the solubility limit (Figure 3) in contrast to Y doping. The atomistic origins of the different behaviors of Sc and Y are explored in Section 8. As a side note, a computationally efficient approach based on the direct solution of the master equation for diffusion that is closely related to kMC can be used in the case of independent particles or by assuming a mean-field type of particle–particle interactions. This approach has also been used successfully to discuss proton diffusion and trapping in Y-doped BaZrO 3 [128,129].

Although kMC simulation and the master equation approach has been successful as mentioned above, it is not without drawbacks. For example, proton–proton interaction as well as the correlated motion of protons, which are expected to have an impact for the heavily doped oxides with high proton content, cannot be considered naturally in contrast to MD (we do note that there are efforts to effectively build some parts of it in to the kMC model [130]). Also, relying on harmonic transition state theory does not work when there is a temperature-dependent structural phase transition caused by soft phonons [131] (*i.e*. when there is strong anharmonicity); this is often the case for perovskite oxides that are cubic at operating temperatures but the cubic structure is unstable at 0 K where ΔEa are calculated using the NEB method. Moreover, kMC is inefficient when there are mixtures of fast and slow processes, since slow processes are seldom selected according to Eq (33). In this regard, an important process in proton conducting oxides is the calcination and sintering processes, *i.e*. equilibration of oxygen vacancy and cation positions which determines the dopant configurations. This equilibration will be very hard to achieve with kMC simulation because cation migration is magnitudes slower than anion migration, and kMC algorithm will almost always select anion migration with very short time steps. Because of this, kMC works in the past have mostly considered a fixed random distribution of dopants, although details of the network topology of dopants should impact the diffusion behavior. In the following, we introduce some of our recent computational works aiming to tackle these issues (Sections 8-10).

## 6. Computational materials discovery for proton-conducting oxides

Traditionally, researchers have explored and developed novel proton-conducting oxides through a process of trial and error. Although it is possible to activate proton conduction by selecting specific structures and combinations of host-dopants that hydrate, the vast number of possible combinations, which is in the millions, poses significant challenges for researchers.

Computations have been utilized mainly for understanding the proton incorporation and diffusion mechanisms. Recent improvements in computing power and the rise of ML have opened up the possibility of efficiently searching for new materials. However, no new materials have been found for proton conductors. This is because proton conduction is a function through complex lattice defects in materials, which have not been successfully incorporated into ML and computations. In this section, we review our recent two successful examples of proton-conducting materials discovery utilizing ML and computations incorporating the fundamental knowledge of point defects.

### 6.1. Traditional materials discovery for proton-conducting oxides: experiments and computations

Proton-conducting oxides have traditionally been developed based on trial-and-error experiments relying on exploration in the analogous system or researchers’ intuition. This is because predicting and controlling the emergence of proton conduction in oxides because the multiple factors impact on whether the hydration reaction and fast proton diffusion is simultaneously satisfied as summarized in Sections 3 and 4. Since the discovery of proton conduction in acceptor-doped SrCeO3 perovskite in 1981 [18], researchers have extensively studied various perovskite hosts with different combinations of A- and B-sites. The presence of proton conductivity has been experimentally confirmed in acceptor-doped LiNbO3 [90,132], KTaO3 [133], NaTaO3 [134], CaZrO3 [37,135–138], CaHfO3 [139,140], CaSnO3 [37], SrTiO3 [141,142], SrZrO3 [136,143–145], SrHfO3 [146], SrCeO3 [18,136], BaTiO3 [41,43,147,148], BaZrO3 [24,96], BaHfO3 [149,150], BaCeO3 [18,136,151,152], BaSnO3 [47,48,153–155], BaPrO3 [156,157], BaTbO3 [14], BaThO3 [14], BaCa1/3Nb2/3O3 [158,159], LaAlO3 [50], LaGaO3 [160], LaInO3 [50,161], LaScO3 [50,161], LaYO3 [50,162] LaErO3 [163,164], LaLuO3 [161], and LaYbO3 [50]. Among them, the BaCeO3 system with the largest lattice volume exhibit the highest proton conductivity while the chemical stability is not satisfactory [165,166]. The solid solution of BaCeO3 and stable and fast proton-conducting BaZrO3 system were explored [165], and BaZr0.1Ce0.7Y0.1Yb0.1O3−δ is developed [167], which is frequently used as electrolyte materials for the proton-conducting ceramics fuel cells [168–180]. This development has followed the design principle for faster proton-conducting oxides: by selecting larger host cations and acceptor dopants in the perovskite structure, the lattice volume has been increased (Section 3.1.3).

Non-perovskite proton-conducting oxides have also been explored through trial-and-error experiments. However, the number of reported materials is much smaller than that of perovskites [25]. Previous studies on non-perovskite oxides have been confined to three cubic and eight non-cubic crystal structures, mainly rare-earth-based oxides [181–188]. Among them, pyrochlore [182], ordered-fluorite [185], and weberite [187] are similar to perovskite with respect to BO6 octahedra sharing their corners. The main difference is in the smaller coordination number of the other cation, which is 8 for pyrochlore and 6 for ordered-fluorite and weberite, compared with 12 for perovskite. Other non-perovskite proton-conducting oxides, including monazite [183,188], fergusonite [184], β-K2SO4 type [181], and eulytite [186], have isolated BO4 tetrahedra rather than BO6 octahedra. The structural rigidity of non-perovskites is characterized by the smaller coordination number of cation, edge- or face-shared cation-oxygen octahedra, rigid tetrahedra, and combinations of these, restricting changes in valence, size, and oxygen loss. These complex structural characteristics can be the reason why non-perovskite proton-conducting oxides are scarce. To activate proton conduction in non-perovskite oxides, it is important to find acceptor dopants that can be doped in these rigid structures.

As explained in Section 5, previous computational studies on proton-conducting oxides have been primarily focused on understanding the energetics of defect formation, defect interactions, and proton diffusion in perovskite structure [20,38,82]. Recently, high-throughput theoretical calculations have been applied to proton-conducting oxides in order to broaden the compositional and structural space to be explored [189–191]. For example, Balachandran et al. calculated formation energies of substitutional Y, oxygen vacancy, and interstitial hydrogen in ≈80 cubic perovskites [189], and Wisesa et al. calculated energy barriers of proton migration in 41 host structures using classical force field calculations [191]. These calculations provided insights into promising candidates of proton-conducting oxides, but the scope of defect calculations was limited to specific cases, especially for acceptor dopants. Resolving this issue is essential for discovering *synthesizable* materials by computationally driven approaches.

### 6.2. Machine learning approach: materials discovery for proton conductors using experimental data

**Figure 5.**
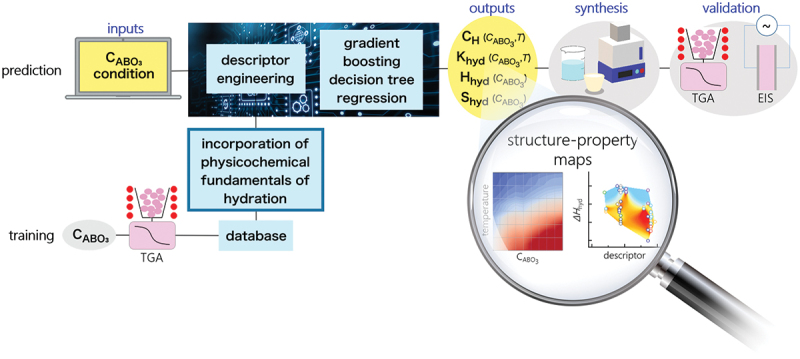
Scheme for exploring and discovering proton-conducting perovskite based on physicochmeical insights of hydration and machine learning. Reprinted with permission from ACS energy lett. 2021, 6, 8, 2985–2992. Copyright 2021 American chemical society.

In this section, we explain an example of materials exploration and discovery for proton conductors by utilizing experimental data and physicochemical knowledge of hydration in ML modeling [35]. Figure 5 shows a workflow of our scheme. The input data are experimental data of proton concentration, CH, determined by thermogravimetry in 65 perovskite oxides (761 experimental data) along with the experimental conditions. The outputs are the proton concentration of hypothetical 8613 chemical compositions (about 100,000 data) under the water partial pressure of 0.02 atm. The selection of one candidate out of 8613 composition was carried out utilizing the structure-property maps, which assist the selection based on the material’s properties and prediction accuracy in unknown materials. Finally, the selected material, Sc-doped SrSnO3, was synthesized, and proton concentration and conductivity were evaluated by the experiments. This scheme demonstrates the power for accelerating the development of proton-conducting perovskites beyond known materials as evident in the discovery by the first attempt. In this section, we explain the details that we took care of and the idea for establishing a good prediction model.

#### 6.2.1. Input training dataset for machine learning

Input training dataset is experimental data of proton concentrations determined by thermogravimetry. We selected the proton concentration of perovskite oxide as a target function due to its high reproducibility. We did not choose the proton conductivity as the target variable because it is more sensitive to microstructure (the density of grain boundaries) influenced by parameters for materials processing.

The selection of reliable data for training dataset is crucial for establishing a reliable predicting model [192], especially for a prediction model using small data. The data for 43 compounds were curated from the literature. Additional 22 compounds were synthesized and evaluated by ourselves. The reliable data is used as the training data to improve the prediction capability. Here, the reliability of data in the literature was mainly judged by two criteria: i) raw data of thermogravimetry was given in the manuscript, and ii) hydration equilibrium can be confirmed in the raw data by the authors. Since the experimental determination of thermodynamic factors is time-consuming, the constructed database from reliable data was small.

We also selected the chemical compositions with a wide variety in chemical space. Although +1/+5 perovskites were not included, the 6 and 8 hosts for +2/+4 and +3/+3 perovskites, respectively, were included in the database with 17 cation species. The acceptor species are doped in respective, and both A- and B-sites were included. This is the result of the inclusion of our minds where a broader chemical space would be beneficial for improving the prediction accuracy since ML prediction is good at interpolative predictions.

#### 6.2.2. Descriptor engineering

The descriptors for ML model were designed by incorporating the accumulated fundamental background summarized in Section 2.4 and 4. The 80 descriptors used in the ML model were defined by the chemical composition, structures, and experimental conditions including synthesis and hydration. All of the descriptors and their ideas behind the design are described below.

##### 6.2.2.1. Descriptors for chemical compositions

The choice of host and dopant chemistry was numerically expressed by the atomic information in terms of atomic weight w, atomic density ρ, melting temperature Tm, first ionization energy E1st, electronegativity χ, ionic radius r. This information is easily available in the literature, so any perovskite oxides can be expressed without any input from experiments, which enables one to explore unknown chemical compositions for development. The distinguishable expression between A- and B-sites, as well as host and dopant elements, was used. Here, we defined ‘dopant’ as an acceptor element. The distinguished expression (rather than the weighted averaged values by the chemical composition) is our intuition in which there are different chemical characters for the basicity of oxide ions coordinated by host and dopant elements.

Weighted average of w, ρ, Tm, E1st, χ and r of A-site elements with its fraction in the A-site
Weighted average of w, ρ, Tm, E1st, χ and r of B-site elements with its fraction in the B-site
Weighted average of w, ρ, Tm, E1st, χ and r of dopant elements with its composition in perovskite
Weighted average of w, ρ, Tm, E1st, χ and r of A-site host elements with its composition in perovskite
Weighted average of w, ρ, Tm, E1st, χ and r of B-site host elements with its composition in perovskite
Sum of descriptors (4) and (5) for each w, ρ, Tm, E1st, χ and r
Ratio of descriptors (1) to (2) for each w, ρ, Tm, E1st, χ and r
(8) Ratio of descriptors (5) to (4) for each w, ρ, Tm, E1st, χ and r
(9) Ratio of descriptors (3) to (4) for each w, ρ, Tm, E1st, χ and r
Ratio of descriptors (3) to (5) for each w, ρ, Tm, E1st, χ and r
Ratio of descriptors (3) to (6) for each w, ρ, Tm, E1st, χ and r
Fraction of dopant elements in A-site
Fraction of dopant elements in B-site
Sum of descriptors (12) and (13)
Fraction of host elements in A-site
Fraction of host elements in B-site
Sum of descriptors (15) and (16)

##### 6.2.2.2. Descriptors for perovskite structures

Features specific to perovskite structure are included in the descriptors.

(18) Tolerance factor

(19) Formula weight of perovskite oxides, Mw

(20) $\frac{Mw}{\left(rArB\right)1/2}$

Tolerance factor, the descriptor (18), is a parameter describing the structural symmetry of perovskite compounds. The structural symmetry was, in fact, reported to be influential on hydration (See Section 3.1.2). The molar density of oxides in descriptor (20) is a descriptor showing the lattice volume of oxide. The descriptor (20), in fact, shows a linear correlation with the lattice volumes in the training dataset. It is proposed as influential on hydration enthalpy [66].

##### 6.2.2.3. Descriptors for experimental conditions in materials synthesis and evaluation

Experimental conditions for materials synthesis and proton concentration measurements were included in the descriptors.

(21) Sintering temperature, Tsinter

(22) Sintering time, tsinter

(23) Temperature, T

(24) Water partial pressure, pH2O

(25) Product of descriptors (21) and (22)

The materials synthesis conditions (sintering temperature and duration time) are also used because they may affect the hydration behavior, especially the local structure and site selectivity of acceptor dopants (See Section 3.1.4.4 and 3.2). The higher sintering temperature makes the distribution of acceptor dopant species closer to random whereas it may deviate the cation nonstoichiometry due to materials evapolation. Variations in hydration behavior have been, in fact, observed in the yttrium-doped barium zirconate synthesized depending on high [36,41] and low sintering temperatures [40,193]. Temperature and water partial pressure of thermogravimetry measurements are variables of proton concentration, as shown in Eq (5).

#### 6.2.3. Target variable engineering to incorporate physical chemistry of hydration into ML model

We incorporated the physicochemical fundamentals of hydration into the ML model by using the target variable CH/Cdopant instead of CH. We made this change because the theoretical maximum of proton concentration is defined by Cdopant (Eqs (1) and (4)), as shown in Figure 2d. By engineering the target variable, the values of proton concentration are normalized between 0 and 1. This normalization provided two benefits: a higher level of confidence for cross-validation (resulting in a smaller root-mean-square error, RMSE), and the ability to predict proton concentration beyond the range of the training dataset, even when using decision tree regressors.

**Figure 6.**
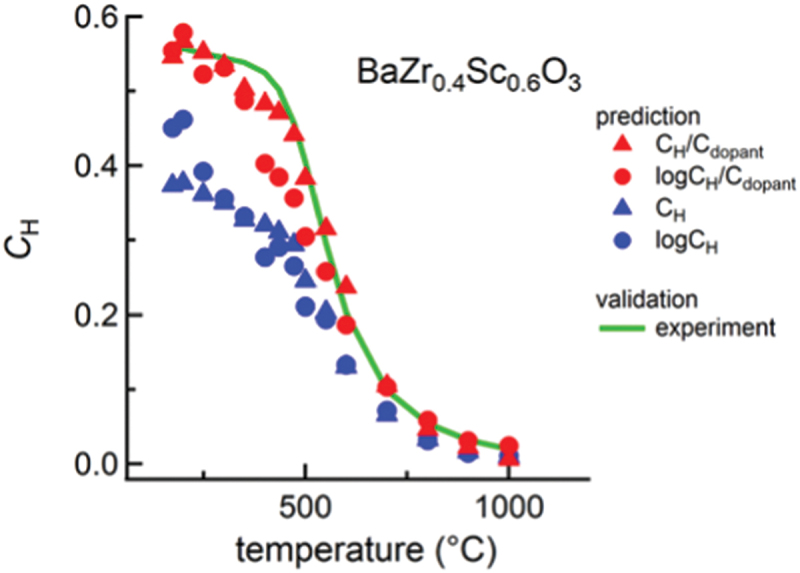
Extrapolation of target values from training data sets using gradient boosting regression. The temperature dependence of proton concentration (*C*_H_) for BaZr0.4Sc0.6O3−δ was predicted. The blue area shows the region beyond the maximum value of proton concentration given by the training database. The solid green line is referred from [24].

We explain the benefits of normalization based on the physical chemistry of hydration using the hydration behavior of BaZr0.4Sc0.6O3−δ, as shown in Figure 6. The material reaches a high proton concentration of 0.55, which is beyond the range (0.38 per formula unit) of proton concentration in our training dataset. According to the definition of decision-tree regressors, which create a decision tree by answering yes/no questions within the training dataset, the ML model cannot provide target values beyond the training data. When using *C*_H_ or log(*C*_H_) as the target variable, the ML model predicts the proton concentration of BaZr0.4Sc0.6O3−δ to 0.4 (blue symbols), which corresponds to the limit of proton concentration in our training dataset. This predicted value is much lower than what we observed in the thermogravimetry measurement (green line). However, by normalizing the proton concentration and selecting the target variables of *C*_H_/*C*_dopant_ or log(*C*_H_/*C*_dopant_) (red symbols), a good match is achieved between the predicted and observed values of proton concentration. By incorporating the physical chemistry of hydration into the ML model through target variable engineering, we were able to overcome the limitation of ML models in predicting target variables beyond the range of the given training dataset. This is crucial for accurately predicting the proton concentration in 8613 hypothetical compounds using a small and sparse training dataset of 65 compounds.

#### 6.2.4. Machine learning model

The ML model used in this study was a gradient boosting regressor (GBR), which falls under the category of decision tree regressors. The GBR was chosen for two main reasons: it demonstrates higher prediction accuracy when compared to random forest, k-nearest neighbors, and kernel-ridge regression, and it has the capability to highlight important descriptors in prediction. It is crucial for us to understand the specific information that the ML model actually learned from the training dataset.

#### 6.2.5. Tools to evaluate what ML model learned from training dataset

Feature importance is a tool that ranks descriptors in modeling and helps us understand which descriptors the ML model learned as important from the training dataset. In Table 2, we have listed the descriptors that were identified as important in our ML model and mentioned in the literature. Out of the 80 descriptors, temperature emerges as the most influential. This finding is consistent with the van’t Hoff equation, Eq (5), which characterizes temperature as a crucial factor in thermally activated processes.

**Table 2.** Descriptor ranking in feature importance of GBR model.

Rank	Descriptor nominated in our ML model	Descriptor proposed in literature	Score
1	Temperature, *T* (°C)	Hydration parameter (S2.2)	0.5877
2	Ratio of dopant to host A-site cation melting point	–	0.0444
3	Average atomic density of host compound	–	0.0419
4	Ratio of dopant to host first ionization energy	Basicity of oxide ion (S3.1.4)	0.0287
5	Tolerance factor	Structural symmetry (S3.1.4)	0.0285
6	Ratio of dopant to host B-site cation in first ionization energy	Basicity of oxide ion (S3.1.4)	0.0276
7	Average first ionization energy of host compound	Basicity of oxide ion (S3.1.4)	0.0207
8	Ratio of A-site cation to B-site cation ionic radius in average	Structural symmetry (S3.1.4)	0.0189
9	Dopant fraction	Prerequisite of Hydration (S2.2)	0.0188
10	Ratio of dopant to host melting point	Oxygen affinity (S7)	0.0170

The high-ranking descriptors from 4th to 10th align with the proposed hydration descriptors found in the literature. The 4th- and 6th-ranked descriptors indicate the type and concentration of the dopant, while the 7th-ranked descriptor specifies the type of host compound. Together, these three descriptors define the combination of the host and dopant. The selection of host-dopant cations determines the 1st ionization energy and electronegativity, factors that influence the basicity of the oxide ion (as discussed in Section 3.1.4.1). Therefore, the 4th-, 6th-, and 7th-ranked descriptors align with the previously proposed descriptor of the oxide ion’s basicity. The 5th-ranked descriptor, tolerance factor, and its related parameter ranked 8th show structural symmetry, which is also a proposed descriptor for hydration (Section 3.1.4.3). The 9th-ranked descriptor, the concentration of the dopant, is the prerequisite for hydration and determines the theoretical maximum of proton concentration (Section 2.2). The 10th ranked descriptor is sensitive to the choice of dopant, specifically the In element which has a low melting temperature. This aligns with the anomalous behavior of hydration observed in In-doped barium zirconate, which can be attributed to the In dopant’s low oxygen affinity (See Section 7 and Ref. [99]). Based on the alignments of descriptors that have been nominated as important in our ML model and also proposed in the literature, we understand that our ML model has successfully learned the physicochemical fundamentals of hydration from the small and sparse training dataset.

**Figure 7.**
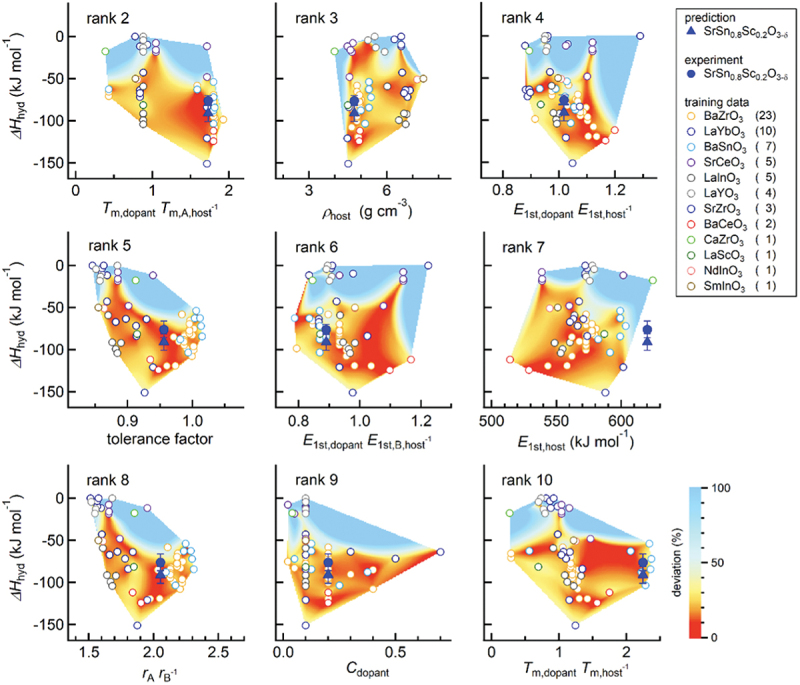
Structure-property maps for hydration against 2nd–10th ranked descriptors. Reprinted with permission from ACS energy lett. 2021, 6, 8, 2985–2992. Copyright 2021 American chemical society.

The structure-property map is another useful tool for evaluating the ML model’s understanding of the training dataset. It illustrates the relationship between the target variable and the descriptor. In Figure 7, the hydration enthalpy of oxides is plotted against the top-ten descriptors, excluding temperature. The training data, represented by open circles, consists of 65 compounds. The colored area represents the interpolative domain in the descriptor space. The colors red and blue indicate high and low levels of confidence in the prediction, respectively. The red region corresponds to deviations in the predicted value of the hydration enthalpy from the experimental value of less than 10%, while the blue region indicates deviations of more than 100% in the cross-validation.

Interestingly, the ML model has identified two new descriptors as the 2nd and 3rd-ranked ones. These descriptors roughly correspond to the +2/+4 and +3/+3 perovskite types. The values of approximately 0.9 and 1.7 in the 2nd-ranked descriptor clearly indicate +3/+3 and +2/+4 perovskites, respectively. The 3rd-ranked descriptor also provides similar information, although differentiating between +2/+4 and +3/+3 perovskites is more subtle. This highlights the ML model’s ability to uncover previously unknown descriptors.

Although the descriptors ranked from 2nd to 10th have some influence, their impact on hydration is not significant. This can be observed from the low scores of feature importance, which range from 0.017 to 0.044 (Table 2). This indicates that the degree of hydration is not determined by a single descriptor, but rather by multiple descriptors. This result agrees with the complexities of oxide hydration, which are extensively discussed in Section 3.1.2.

#### 6.2.6. Selection and discovery of a new proton-conducting oxide based on physicochemical interpretation

ML models, especially decision tree regressors, are effective in predicting target values when they are within the training dataset. This is called interpolative conditions. However, a challenge arises when it comes to identifying new materials. This is because new materials have chemical compositions that do not match the range of known materials. ML models have difficulty in accurately predicting outcomes outside the range of the training dataset (extrapolative prediction). Now, the question is as follows: how can we efficiently choose one candidate for new proton-conducting oxide from 8613 compositions?

Our approach involved actively using the physicochemical interpretation of hydration during the selection process. It is known that high-performance proton-conducting oxides, such as Y- and Sc-doped barium zirconates, have hydration enthalpies of approximately −100 kJmol^−1^ [14,24]. Therefore, we set a screening threshold based on this empirical knowledge (−100 ± 5 kJmol^−1^). As a result, the number of candidates was reduced from 8613 to 551, which represents only 6% of the initial pool. The second threshold we considered was the reliability of the predictions made by the ML model. Since we know that ML prediction is most useful under interpolative conditions, we focused on candidate compositions that fell within the interpolative domains and showed a higher level of confidence for the 2nd- to 4th-ranked descriptors. These compositions are highlighted in red on the structure-property map (Figure 7). As a result of this criterion, we identified five potential hosts consisting of 49 BaCeO_3_, 15 BaZrO_3_, 12 SrZrO_3_, 12 SrSnO_3_, and 5 BaSnO_3_ compounds (93 in total). It is worth noting that SrSnO_3_ is the only host oxide that has not to be reported for hydration and proton conduction. Based on the high-performance results reported in [24], we selected Sc as the dopant from the pool of 12 candidates and 20at%, rather 40at%, for its doping level because it is easier to dissolve Sc into the perovskite phase.

**Figure 8.**
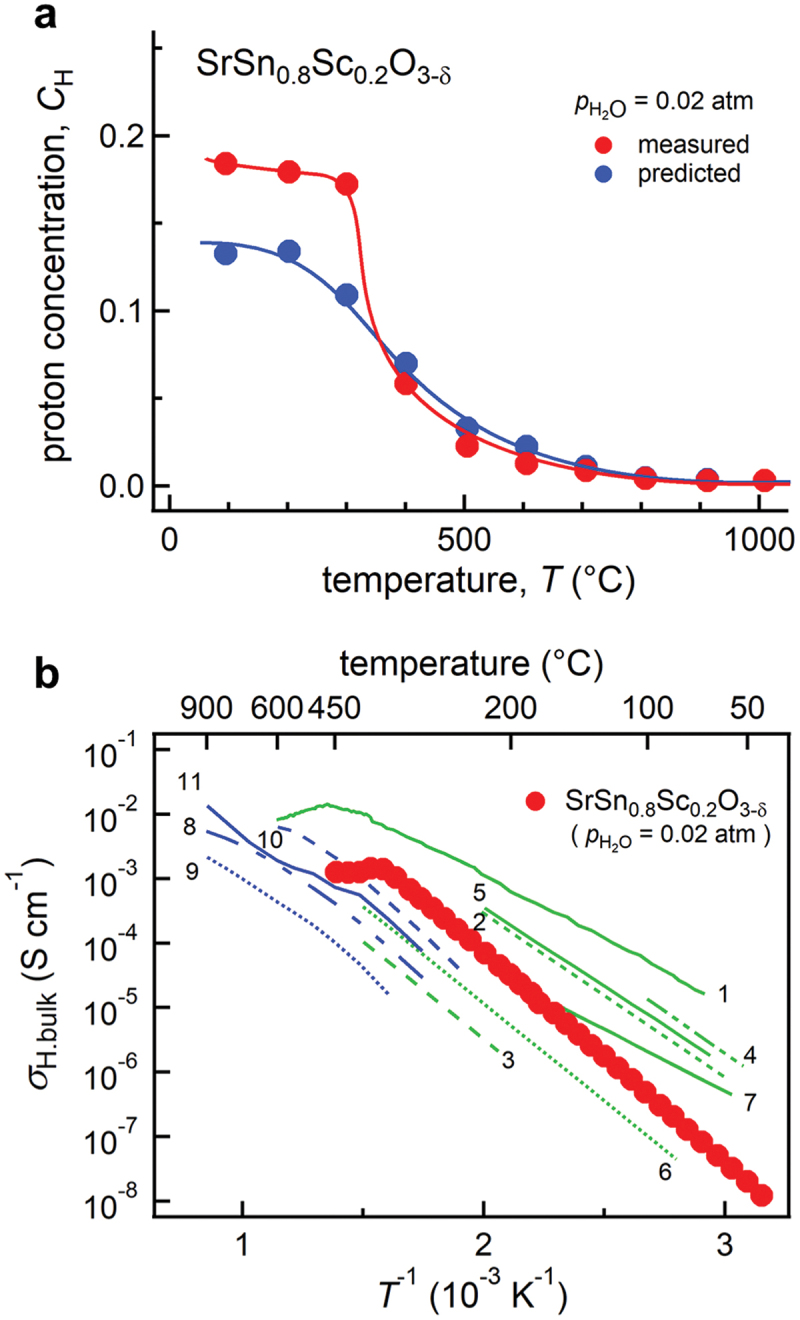
Discovery of proton-conducting perovskites using machine learning model. a) proton incorporation and b) proton conduction in SrSn0.8Sc0.2O3−δ. The red and blue symbols in a) show the measured and predicted values, respectively. The green and blue lines in b) correspond to reported proton conductivities in +2/+4 and +3/+3 perovskites, respectively, with the reference numbers 1: BaZr0.4Sc0.6O3−δ [24], 2:BaCe0.9Y0.1O3−δ [43], 3:BaTi0.95Sc0.05O3−δ [43], 4: BaSn0.5Y0.5O3−δ [43], 5: BaCa0.39Nb0.61O3−δ [43], 6: SrZr0.9Y0.1O3−δ [43], 7: SrTi0.95Sc0.05O3−δ [43], 8: La0.9Ba0.1YbO3−δ [51], 9: La0.9Sr0.1YO3−δ [50], 10: La0.9Sr0.1ScO3−δ [50], 11: La0.9Sr0.1InO3−δ [50]. Reprinted with permission from ACS energy lett. 2021, 6, 8, 2985–2992. Copyright 2021 American chemical society.

The selected composition of SrSn0.8Sc0.2O3−δ clearly demonstrates the proton incorporation as predicted by the ML (Figure 8a) and proton conduction (Figure 8b). The proton concentration at temperatures above 400  ∘C closely matches both the predicted and measured values.

It should be emphasized that SrSn0.8Sc0.2O3−δ was selected as our first choice. The unbiased ML model enables us to study the chemical composition of the B-site cation, moving beyond the conventional 4d Zr [14,24] to the p-block Sn. This showcases the effectiveness of our methodology in expediting the discovery of previously unknown perovskites that conduct protons.

### 6.3. High-throughput ab initio calculations: discovery of non-perovskite proton conductors

**Figure 9.**
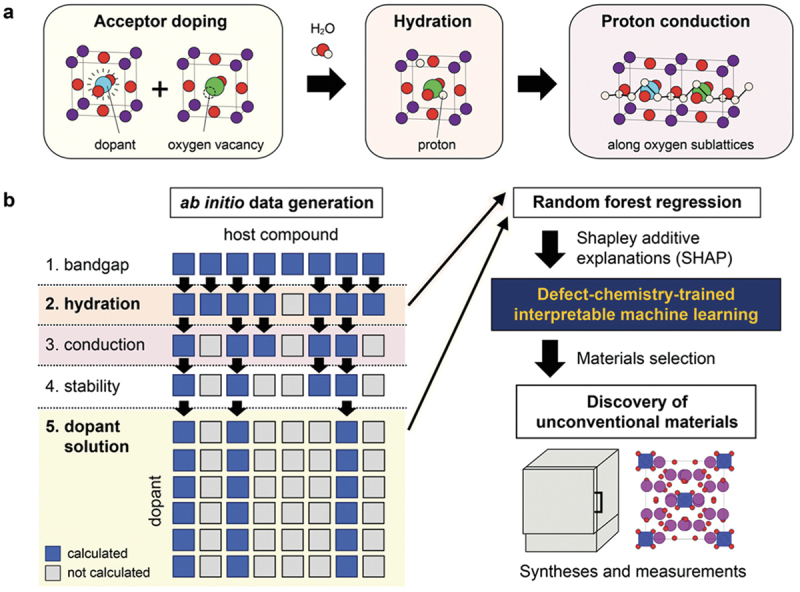
(a) Activation process of proton conduction in oxides. (b) Workflow to explore and discover unconventional proton-conducting oxides. Reprinted under the CC-BY 4.0 license from [28].

As explained in Section 2, defect chemistry is essential to activate proton conduction in oxides. However, experimental data on point defects are not available for unexplored structural and compositional space, hindering the discovery of new proton-conducting oxides. This is where high-throughput calculations come into play. We have recently demonstrated that calculating energies of chemical reactions involving three types of point defects shown in Figure 9, namely acceptor dopant, oxygen vacancy, and interstitial proton, is a straightforward and powerful means of exploring unconventional non-perovskite proton conductors [28].

#### 6.3.1. Computational screening

Our methodology is schematically shown in Figure 9b. This is the sequential *ab initio* calculations for screening promising compounds from vast host-dopant combinations. The Vienna Ab initio Simulation Package (VASP) [194,195] was employed for all *ab initio* calculations. The first four steps are calculations according to the requirements for the host oxide: (1) wide band gap for dominant proton conduction than electronic conduction, (2) ease of hydration via oxygen vacancy, (3) fast proton conduction, and (4) thermodynamic stability, respectively. The step 1 simply calculates the band gap by *ab initio* calculations. The step 2 calculates the hydration energy via an oxide-ion vacancy, requiring a large number of defect calculations including the search of stable proton sites and the formation of oxide-ion vacancies. The step 3 concerns proton conduction, and we simplify this step only to consider the formability of proton conduction pathways based on the stable proton sites and their energies obtained in the step 2, due to the computational cost. The step 4 ensures the thermodynamic stability of the host compound by generating a computational phase diagram. The screened promising host oxides are proceeded to the final step, where dopants are considered. This step exactly calculate the solution energy of dopants, by performing dopant calculations and re-generating a phase diagram containing dopant elements to estimate chemical potentials of constituent elements. The most computationally demanding steps of the sequential computation is those regarding defect calculations, namely step 2 (hydration reaction; vO and Hi) and step 4 (dopant solution; vO and MA), but these must be incorporated to the screening because they are essential for the activation of proton conduction in oxides (Figure 9a). Note that the chemical stability of compounds, for example against CO2 gas, is necessary for proton conductors in practical use, but is not considered in our current methodology.

Automated calculations and pre- and post processing are essential in realizing accelerated materials discovery. The sequential computation relies heavily on in-house shell and Python scripts utilizing Pymatgen [3,196,197] and Spglib [198]. These scripts automate the following tasks: (1) remove compounds with partial site occupancy or overlapped compounds from the target host compounds extracted from Inorganic Crystal Structure Database (ICSD) [199]; (2) set appropriate conditions for *ab initio* calculations based on crystal structure and elements included; (3) generate point defect models with an oxide ion vacancy or a proton introduced, taking symmetry into account; (4) calculate hydration energies from the point defect calculations; (5) determine the presence or absence of proton conduction pathways based on the positions and energies of proton sites using graph theory (similar to the method shown in Section 8); (6) generate point defect models with a dopant introduced; (7) extract compounds included in a phase diagram [3,197], which contains a target host compound and a dopant oxide, from the Materials Project database; (8) determine the chemical potentials of elements at each equilibrium state from re-generated phase diagram [196] and calculate the solution energies of dopants. Building these scripts is practically the most time-consuming task in operation. In addition, the use of supercomputing systems capable of performing large-scale *ab initio* calculations with the generated input files is necessary for exploring promising materials in a realistic time. In our case, supercomputers at the Institute for Solid State Physics, The University of Tokyo (Ohtaka) and at RIKEN Center for Computational Science (Fugaku) were used for more than 3,500 point defect calculations and energy calculations of ∼1,500 compounds for generating phase diagrams.

The hydration energy, Ehyd, and dopant solution energy, Esol were calculated in the screening process based on Eq (4) and (1) as follows:

(36) $$E\mathrm{hyd}=\left\{2E\mathrm{def}(OHO∙)\right\}-\left\{E\mathrm{def}(vO∙∙)+E\mathrm{perf}(OO\times )+E(H2O)\right\},$$

(37) $$E\mathrm{sol}=\left\{E\mathrm{def}(MA\prime )+\mu A+\frac{1}{2}E\mathrm{def}(vO∙∙)+\frac{1}{2}\mu O\right\}-\left\{E\mathrm{perf}(AA\times )+\mu M+\frac{1}{2}E\mathrm{perf}(OO\times )\right\},$$

where Eperf(OO×) and Eperf(AA×) are the energy of a perfect supercell of the host compound, Edef(OHO∙), Edef(vO∙∙), and Edef(MA′) are the energies of a charged supercell of a host compound containing a proton, an oxide-ion vacancy, and a dopant M at the site of A cation, E(H2O) is the energy of a water molecule, and μA, μM, and μO are the chemical potentials of A, M, and O, respectively. Here, the symbol E is used instead of the symbol G, as only the potential energy at 0 K is considered. The term $\left(\mu A+\frac{1}{2}\mu O-\mu M\right)$ that appears in Eq (37) is obtained from the energies of competing phases in a manner like Eq (22). For example, the term $\left(\mu \mathrm{Zr}+\frac{1}{2}\mu O-\mu \mathrm{Sc}\right)$ for Sc-doped BaZrO3 that coexists with BaSc2O4 and Ba3Zr2O7 can be obtained as

(38) $$\mu \mathrm{Zr}+\frac{1}{2}\mu O-\mu \mathrm{Sc}=2E(\mathrm{BaZr}O3)-\frac{1}{2}E(\mathrm{BaS}c2O4)-\frac{1}{2}E(Ba3Zr2O7).$$

Considering competing phases in a phase diagram instead of assuming a reaction with simple metal oxides is essential to accurately calculate the dopant solution energy, as explained in Section 6.3.2. According to Eq (21) for defect formation energy, Ehyd and Esol can be also expressed as follows:

(39) Ehyd=2ΔEdef(OHO∙)−ΔEdef(vO∙∙),

(40) $$E\mathrm{sol}=\Delta E\mathrm{def}(MA\prime )+\frac{1}{2}\Delta E\mathrm{def}(vO∙∙).$$

Note that vibrational effects and configurational entropy explained in Section 5.1 are not taken into account in the sequential computation due to the computational cost.

#### 6.3.2. Examples of materials discovery

**Figure 10.**
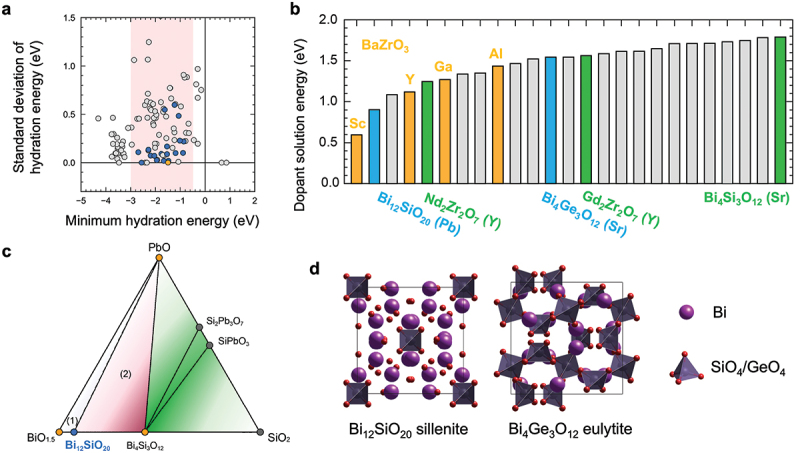
(a) Minimum hydration energy and standard deviation of hydration energies for multiple proton sites identified by calculations. Grey and blue circles show the results of non-perovskite host oxides with and without proton conduction pathways. Yellow circle shows the result of BaZrO3 perovskite as a reference. (b) Calculated dopant solution energies of each host-dopant combination. Those having low dopant solution energies were shown in this figure from total 200 combinations calculated. Yellow, green, blue, and grey bars shows the results of BaZrO3 perovskite, known non-perovskite proton-conducting oxides, promising candidates of proton-conducting oxides, and others. An element in parentheses indicates a dopant. (c) Computed phase diagram of quasi-ternary BiO 1.5-SiO 2-PbO system for estimating dopant solution energy of Pb-doped Bi12SiO20 sillenite. There are two equilibrium states to be considered, namely (1) Bi-rich and (2) Si-rich conditions. The dopant solution energy depends on the equilibrium state. (d) Crystal structures of two promising candidates of non-perovskite proton-conducting oxides identified from the sequential computation. Reproduced from [28] under the CC-BY 4.0 license.

To discover new and unconventional proton-conducting oxides, we applied the developed screening scheme to 301 cubic oxides taken from the ICSD, which have 1689 possible combinations with dopants. The step 2 of the screening showed that various non-perovskite oxides have moderately negative hydration energies comparable to BaZrO3 (red region in Figure 10a). This means that many oxides can be hydrated regardless of constituent elements and crystal structures, if the prerequisite of hydration, *i.e*. oxide ion vacancy, exists in the host oxide. However, the step 3 revealed that forming proton conduction pathways is difficult for most compounds, including major crystal structures such as garnet. This is partly due to the existence of sites with high energy that protons cannot pass through, as implied by the large standard deviation of hydration energies between multiple proton sites in Figure 10a. Only the compound shown in blue in Figure 10a passed the step 3, which are a part of pyrochlores, spinels, and exotic crystal structures containing heavy elements such as Rb, Cs, Sn, and Bi, except for perovskites.

Dopant solution energies for these host compounds were calculated in the final screening step. This step successfully showed that the host-dopant combinations that have been known as proton conductors, *i.e*. Sc- and Y-doped BaZrO3 perovskite [24,40], Y-doped Nd2Zr2O7 and Gd2Zr2O7 pyrochlores [200], and Sr-doped Bi4Si3O12 eulytite [186], have relatively low dopant solution energy between 200 host-dopant combinations calculated (Figure 10b). We emphasize that, when estimating dopant solution energy, it is essential to calculate phase diagrams to determine the chemical potentials as accurately as possible from the equilibrium states (Figure 10c). In our preliminary study, with the chemical potentials from the energies of simple binary oxides, the order of the dopant solution energy and the experimental solubility limit of Sc, Y, Ga, and Al (∼59% [24,201], ∼50% [70], ∼15% [202], and ∼15% [202]) in BaZrO3 did not correspond, contrary to Figure 10b. The dopant solution energy calculated with the phase diagrams significantly narrowed down the number of candidates, indicating the difficulty in forming oxide-ion vacancies in non-perovskite structures. Finally, Pb-doped Bi12SiO20 sillenite and Sr-doped Bi4Ge3O12 eulytite were identified as promising cancdidates (Figure 10d).

Proton conduction in the screened oxides were demonstrated by experiments. The dopant solutions in Bi12SiO20 and Bi4Ge3O12 were successfully demonstrated by X-ray diffractometry, with sillenite and eulytite as main phases, and gradual decreases in lattice constants with increasing dopant content. AC impedance spectroscopy and isotope exchange experiments further revealed the proton conduction in the Pb-doped Bi12SiO20 and Sr-doped Bi4Ge3O12, with moderate conductivities among non-perovskite proton-conducting oxides. Of these two, Pb-doped Bi12SiO20 is the first proton-conducting oxide, both as a sillenite structure and as composed solely of groups 14 and 15 cations. The success of the first trials of synthesis demonstrated our screening methodology, which incorporates the stability and function of point defects, captures the essence of proton-conducting oxides. Further improvements in screening criteria, *e.g*. estimation of energy barrier during proton migration, and an expanded chemical and structural space of exploration would facilitate the development of fast proton-conducting oxides that are not limited to specific crystal structures and/or constituent elements.

#### 6.3.3. Machine learning with generated computational database and descriptors

General and fundamental understanding obtained using ML models assisted in selecting Pb-doped Bi12SiO20 and Sr-doped Bi4Ge3O12 as experimental targets from a large amount of computational data described above (Figure 9b). In the screening process, we collected materials’ data as wide as possible by setting relatively loose criteria within the limits of available computational resources. As a result, 80 hydration energies for the host compounds with 18 crystal structures, and 200 dopant solution energies with 28 compounds and 11 crystal structures were obtained. The number of data is not large enough to cover any materials, but it is at least more general in the compositional and structural space than a database focused on a specific crystal structure such as perovskite. Using the database, random forest models for hydration and dopant solution energies were constructed using scikit-learn [203]. Shapley additive explanations (SHAP) [29] was applied to the models to interpret the important features (descriptors) on these reaction energies.

After many trials by hand, only four and seven descriptors were used for creating random-forest models of hydration and dopant solution energies. The descriptors are mean coordination number of cations, volume per atom, mean ionic radius of cations, and mean electronegativity of cations for the hydration-energy model, and difference in ionic radius and electronegativity between dopant and substituted host cations, and reaction energy of the host compound for the dopant-solution model, in addition to those used for the hydration energy model. Keeping the number of descriptors to a minimum while ensuring sufficient prediction accuracy improves the interpretability of the models. Here, the constructed models shows the root mean square errors (RMSEs) of 0.19 and 0.11 eV for the training and test datasets in hydration energy, and 0.27 and 0.26 eV in dopant solution energy.

#### 6.3.4. Interpretation and selection of experimental targets

**Figure 11.**
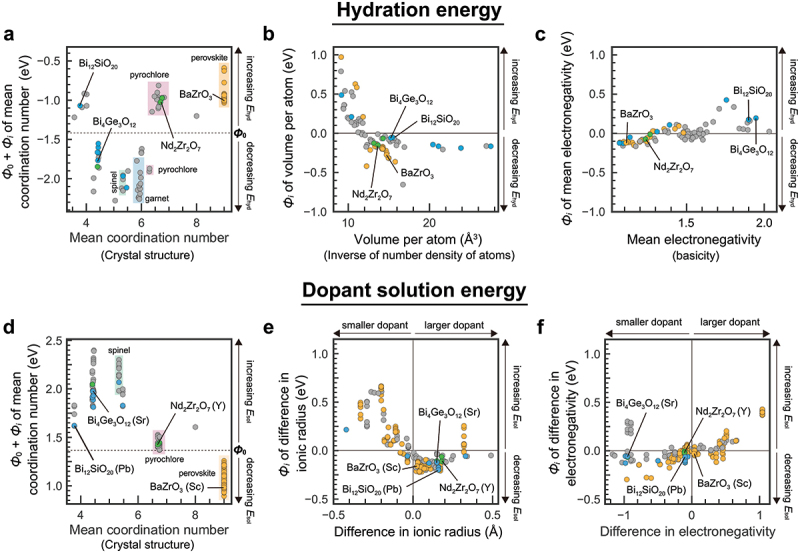
Impact of top-three descriptors that affect (a-c) hydration energy and (d–e) dopant solution energy in the constructed random-forest models (SHAP values ϕi). (a,d) mean coordination number. (b) Volume per atom. (c) Mean electronegativity. (e, f) difference in (e) ionic radius and (f) electronegativity between dopant and substituted host cation. The base value ϕ0 is added to ϕi of the most impactful feature, *i.e*. mean coordination number, in (a) and (d). Perovskites, reported non-perovskite proton-conducting oxides, promising non-perovskite oxides predicted by computation, and other non-perovskite oxides are indicated by yellow, green, blue, and gray circles, respectively. Reproduced from [28] under the CC-BY 4.0 license.

SHAP analysis [29] on the constructed random-forest models provides quantitative insights on how each descriptor affects the hydration and dopant solution energies. In the SHAP analysis, hydration or dopant solution energy E can be expressed as the summation of contributions from descriptors,

(41) $$E=\phi 0+\sum_{i=1}^{N}\phi i(xi),$$

where xi is the value of descriptor i, N is the number of descriptors, and ϕ0 is the average value of hydration or dopant solution energy over the training dataset (base value). Figure 11 shows the SHAP values ϕi of top three descriptors i that affect the hydration and dopant solution energies. Mean coordination number of cations, which serves as an identifier of crystal structure, is the most impactful descriptor both for the hydration and dopant solution energies. Notably, perovskite is only the structure that decrease dopant solution energy between cubic structures and shows moderately negative hydration energy around −1 eV, which are both benefits for activating proton conduction. The second and third most impactful descriptors on hydration is volume per atom and mean electronegativity of cations, which serve as the indicators of structural openness and basicity, respectively. As shown in Figures 11b,c, hydration is more likely to occur as the structure becomes more open or less basic, agreeing with the empirical rules found for perovskites [14]. A quantitative insight from this model, which includes a wider range of crystal structures, is that structural features are more important than conventional descriptor of basicity for hydration, as the SHAP values of the structural features are larger than that of the mean electronegativity of cations. For dopant solution energy, the second and third most impactful descriptors are the indicators of host-dopant compatibility, namely difference in ionic radius and electronegativity between dopant and substituted host cation. As the empirical rule, the closer the size of dopant and host cation is, the more likely dopant solution occurs (Figure 11e). It was quantitatively shown that slightly larger ionic radius of dopant compared with host cation (up to 0.3 Å) facilitates dopant dissolution into oxides. Lower electronegativity of dopant also enhance dopant dissolution, which is also physically reasonable. The trends in the ML models follow empirical rules, demonstrating the validity of the models and providing quantitative and more general insights into the impact of structural and chemical features on the reaction energies involving point defects.

**Figure 12.**
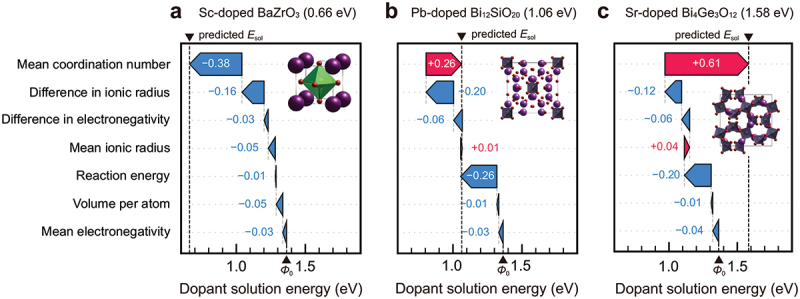
Descriptor contributions (SHAP values) to dopant solution energies derived from the random-forest model. (a) Sc-doped BaZrO3 perovskite. (b) Pb-doped Bi12SiO20 sillenite. (c) Sr-doped Bi4Ge3O12 eulytite. Reprinted from [28] under the CC-BY 4.0 license.

ML models can also provide why the screened compounds, which are experimentally unknown for proton conduction, are promising candidates. Figure 12 shows the SHAP values of each descriptor for Sc-doped BaZrO3 (as a reference), Pb-doped Bi12SiO20, and Sr-doped Bi4Ge3O12. In Sc-doped BaZrO3, all descriptors, especially mean coordination number of cations (crystal structure), decrease the dopant solution energy, resulting in the low value of 0.66 eV (Figure 12a). The low value is consistent with the experimental fact that the solubility limit of Sc in BaZrO3 is as high as ∼60% [24]. On the other hand, in Bi12SiO20 and Bi4Ge3O12, the SHAP value of mean coordination number of cations (crystal structure) largely increases the dopant solution energy, clearly showing the disadvantage of non-perovskite structures. However, the compatibility between dopant and host cation (difference in ionic radius and electronegativity) and reaction energy (thermodynamic stability) of host compound significantly decrease their dopant solution energy (Figures 12b,c). These features yield acceptable dopant solution energies of 1.06 eV for Pb-doped Bi12SiO20 and 1.58 eV for Sr-doped Bi4Ge3O12. This physicochemical interpretation enabled the authors to synthesize and measure the screened candidate compounds with confidence.

## 7. Oxygen affinity: a hidden parameter linking hydration, proton-dopant association, and proton conductivity

Hydration energy and proton-dopant association energy have been a major concern that relates to proton diffusion and conduction in perovskite oxides. Researchers have tried to control these parameters independently. Recently, we reinvestigated the defect chemistry of hydration reactions and found that the hydration reaction can be decomposed into several elementary defect reactions [99]. Especially, we discovered ‘oxygen affinity’, which can only be assessed by computations at present, as a decisive factor that correlates hydration with proton-dopant association and the proton conductivity. We define ‘oxygen affinity’ as the energy change of oxidation reaction where a vacant site is filled by an oxide ion as shown in Eq (6). In the following, we summarize our findings regarding this ‘hidden’ parameter linking hydration, proton-dopant association, and proton conductivity, as revealed from calculations for BaZrO3.

**Figure 13.**
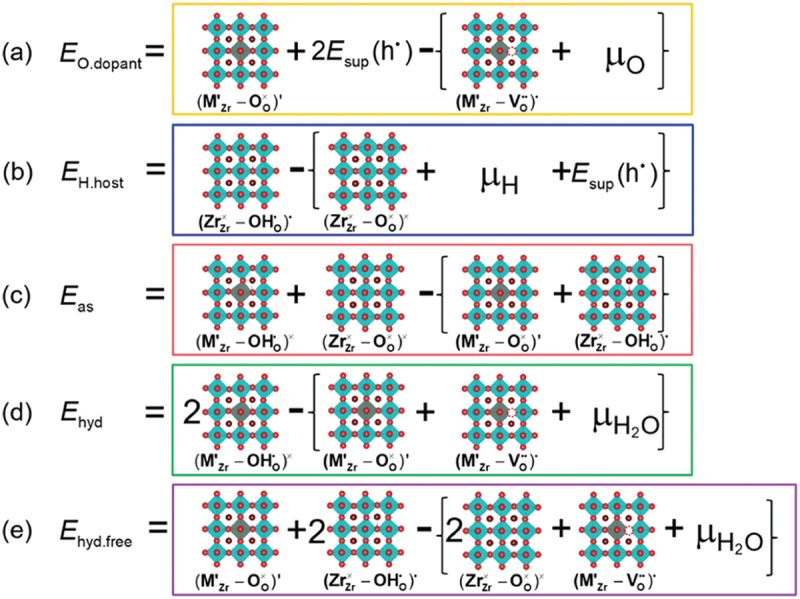
Schematic representations of energy terms: (a) oxygen affinity, EO.dopant, (b) hydrogen affinity, EH.host, (c) association energy, Eas, (d) hydration energy, Ehyd, and (e) hydration energy of free (nontrapped) protons, Ehyd.free. The colors of the right-hand-side boxes correspond to the colors of the arrows in Figure 15. Reprinted with permission from Chem Mater. 2020, 32, 7292-7300. Copyright 2020 American chemical society.

Energies of elemental processes of the hydration reaction can be evaluated using *ab initio* calculations. The objective energy terms in our analytical framework and their schematic representations are listed in Figure 13. These are the hydration energy, Ehyd, the proton-dopant association energy, Eas, the oxygen affinity, EO.dopant, the hydrogen affinity, EH.host, and the hydration energy of free (nontrapped) protons, Ehyd.free. In this section, Eas is computed as the energy difference between the most stable proton configuration associated with an acceptor dopant and an isolated proton. The hydrogen affinity, EH.host, is a property of the undoped host BaZrO3. Eas and EH.host correspond to the energy changes per proton. It should be noted that EO.dopant considers the oxidation of an oxide ion vacancy neighboring acceptor dopant and delocalized hole formation. EO.dopant depends on the type of dopant. In the evaluation of Ehyd and Ehyd.free, a hydration reaction, where two protons are incorporated into the lattice of BaZrO3 from one oxide ion vacancy, are considered. Details of the formula derivation of these energy terms can be found in Ref. [99]. In our analytical framework, the symbol E is used instead of the symbol G in Section 5.1, as the terms pV and entropy in free energy are not considered.

**Figure 14.**
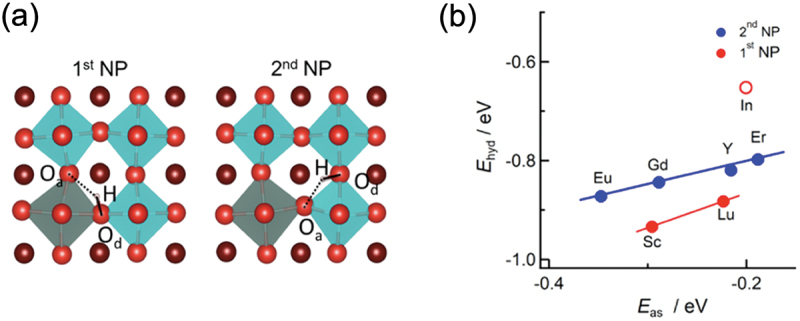
(a) Local structures around a dopant and proton on 1st and 2nd nearest proton (NP) sites. (b) Hydration energy (Ehyd) as a function of proton-dopant association energy (Eas). 1st and 2nd NP in (b) indicate dopants where a proton is most energetically stable at the 1st and 2nd NP sites, respectively. Adapted with permission from Chem Mater. 2020, 32, 7292-7300. Copyright 2020 American chemical society.

In acceptor-doped systems, hydroxyl groups were assumed to be incorporated into oxygen vacancy sites associated near trivalent dopants. Similarly, protons were assumed to be incorporated into oxygen sites near the dopants. Therefore, Ehyd was determined by considering defect configurations with the lowest energy before and after hydration. This is a more accurate treatment of hydration than Section 6.3, which considers hydration in undoped hosts due to the computational cost. For the calculation of the proton-dopant association energy, Eas, we used the most stable configuration of the proton as in the case of the hydration energy. For all dopants, the association energy calculated in BaZrO3 is negatively larger when the proton is close to the dopant. The 1st nearest proton site is the most stable for smaller dopants Sc, In, and Lu, while the 2nd nearest proton site is the most stable for larger dopants Er, Y, Gd, and Eu (Figure 14a).

Calculated hydration energies showed a trade-off relationship with proton-dopant association energies (Figure 14b). The greater the hydration energy, the larger the association energy, meaning that a trade-off relationship between the proton concentration and proton diffusivity depending on the dopant species. More negative hydration and less negative association energies would be ideal to maximize the proton concentration and diffusivity simultaneously. Such materials are located in the bottom-right region of Figure 14b.

The hydration reaction, with energy Ehyd, can be conceptualized as the summation of three reactions in Figure 13, by combining and rearranging the energy components of each term on the right-hand side of Figure 13:

(42) Ehyd=EO.dopant+2EH.host+2Eas.

The first is oxidation, where a trapped oxygen vacancy is filled by an oxygen atom and two electron holes are generated. The oxygen affinity for a given dopant EO.dopant corresponds to the energy of this reaction (Figure 13a). The second reaction is hydrogenation, where gaseous hydrogen is incorporated as proton at oxygen sites far from the dopant, with the consumption of a hole. This reaction can be regarded as the affinity of the perfect crystal to hydrogen, *i.e*. hydrogen affinity EH.host (Figure 13b). The third reaction is proton-dopant association, where Eas was released when an isolated proton gets trapped near a dopant (Figure 13c). As the proton trapping site is dependent on the size of the dopant, the degree of local lattice distortion caused by the dopant should be the dominant factor for Eas.

**Figure 15.**
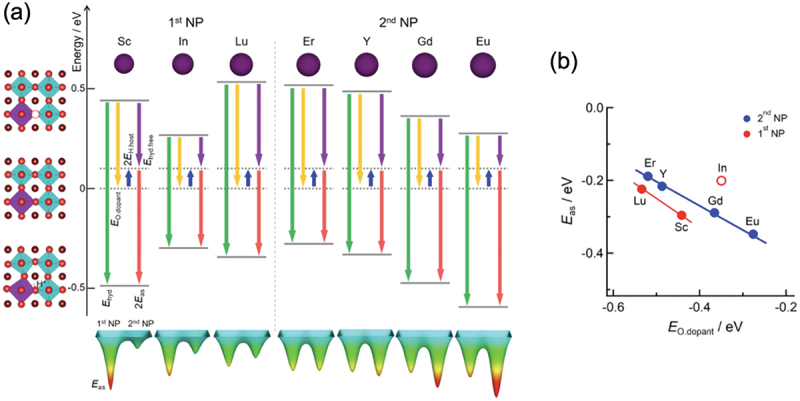
(a) Relationship between hydration energies Ehyd (green), oxygen affinities EO.dopant (yellow), hydrogen affinities EH.host (blue), proton-dopant association energies Eas (red), and hydration energies for creating trap-free protons Ehyd.free. The results of dopants are shown in order of increasing ionic radius from left to right. At the bottom of figure, the proton-dopant association energy wells for a proton occupying 1st and 2nd nearst proton sites to the dopant are visualized as the contour plots. Red portions mean deep proton trapping. (b) Eas as a function of EO.dopant. 1st and 2nd NP indicate dopants where a proton is most energetically stable at the 1st and 2nd NP sites, respectively. Adopted with permission from Chem Mater. 2020, 32, 7292-7300. Copyright 2020 American chemical society.

The relationship between Ehyd and three virtual reactions in Eq (42) is schematically shown in Figure 15a. The three main states in the hydration process are also displayed on the left-hand side of Figure 15a. In this figure, energy states of oxidized acceptor solid solution where oxygen vacancy sites are occupied by oxide ions are set to be 0 eV as a reference state in the hydration reaction process. The topmost is the initial state after acceptor doping, with the introduced oxygen vacancies occupying the most stable sites next to the dopant. The middle structure corresponds to the virtual oxidation step acting to stabilize the acceptor-doped system (yellow arrows). Our *ab initio* calculation showed that EH.host is only 0.050 eV, indicating that hydrogenation slightly destabilizes the system (upward pointing blue arrows). The final structure at the bottom is after proton trapping (proton-dopant association), which largely decreases the total energy (red arrows), in some cases to a strongly negative Ehyd.

For proton incorporation, the hydration energy of the material (green arrows in Figure 15a) needs to be more negative than the oxygen affinity (yellow arrows in Figure 15a). In such a case, the oxygen vacancies are more energetically favorable to be occupied by hydroxyl groups than by oxide ions. This implies that proton incorporation is more likely to occur than electron hole formation. The stronger the proton-dopant association (large red arrows), the more strongly negative hydration energy, which is required to produce a high proton concentration. Such a relationship can be seen in Figure 14b. However, a large negative trapping energy is undesirable because it also means that the mobility of incorporated protons is low. The most desirable situation is that a material has a large hydration energy for proton incorporation without trapping. The hypothetical hydration energy for creating such trap-free protons, Ehyd.free, can be calculated as the summation of EO.dopant and 2EH.host (yellow and blue arrows in Figure 15a):

(43) Ehyd.free=EO.dopant+2EH.host,

which is shown in Figure 15a as purple arrows. Eqs (42) and (43) show that only two material parameters, Eas and EO.dopant, can be tuned by dopant selection to develop a faster proton-conducting oxides. Since the main contribution to proton conductivity is mobile protons that are not trapped in the vicinity of dopants, a material with a large negative Ehyd.free and hence large oxygen affinity EO.dopant should exhibit high proton conductivity. Because EH.host is independent of the type of dopant, EO.dopant can serve as a proxy for Ehyd.free to identify doped systems with high concentrations of mobile protons, for a given host material.

Figure 15b shows the inverse linear correlation between Eas and EO.dopant. Interestingly, even though the association and oxidation reactions can be considered separately, this result suggests that the choice of dopant for a given host material does not control these energy terms independently. The ideal material should have more negative Ehyd.free and less negative Eas, and such a condition can be satisfied when the oxygen affinity is strongly negative, according to Eq (43) and Figure 15b. Thus, EO.dopant appears to be the most reliable predictor of proton conductivity, and suggests that Er, followed by Lu, is the dopant that provides the highest proton conductivity for BaZrO3. The validity of this theoretical framework was demonstrated by experiments as reported in Ref. [99].

The above new theoretical framework and its experimental demonstration show that the oxygen affinity near a dopant, EO.dopant, provides the most reliable measure for predicting the degree of proton conduction in BaZrO3. The reason for this is that the oxygen affinity is directly related to the hydration energy for trap-free protons, Ehyd.free, and can be used to identify dopants capable of incorporating high concentrations of mobile protons. The correlation between oxygen affinity and proton conductivity is not restricted to a particular host crystal as is theoretically evident from Eq (43). Thus, this concept is expected to be applicable to the search for suitable acceptor-dopant for various proton-conducting oxides. Also, if the host material is also the target of the search, the hydrogen affinity, EH.host, becomes the variable that controls Ehyd.free in addition to EO.dopant of Eq (43). In other words, host materials with a largely negative EH.host should be searched for. Incorporating this theoretical framework into the computational screening as presented in Section 6.3 would make the search for faster proton-conducting oxides more efficient.

## 8. Machine learning and graph theory analysis to understand proton diffusion in oxides

An important question mentioned in above sections is why Y and Sc dopants differently influence proton diffusivity with increasing dopant concentration as shown in Figure 3. Here, we focus on octahedral tilts and distortions for understanding this difference in heavily doped systems up to 25 at% [204]. In the past, *ab initio* calculations have been performed to understand proton diffusion in oxides, specifically in perovskite oxides as mentioned in Section 5, but the main focus of these calculations was on proton-dopant association enthalpies in relation with dopant-proton configurations and/or hydrogen bonding. Local structures, related to hydrogen bonding, have also been investigated in a dilute solution of dopant in the barium zirconate. The dependence of proton diffusivity on dopant species and its concentration, however, has not been clearly explained in barium zirconate, specifically in terms of local distortions. MD and kMC studies mentioned in Section 5 have also mostly overlooked this aspect. Here, we review our recent works employing graph theory analysis to effectively search for proton diffusion pathways and crystal structures along those pathways from all atomic configurations. ML regression is then used on these pathways to extract important descriptors for proton diffusivity in terms of local tilts and distortions.

### 8.1. Graph theory pathway search and descriptor extraction

**Figure 16.**
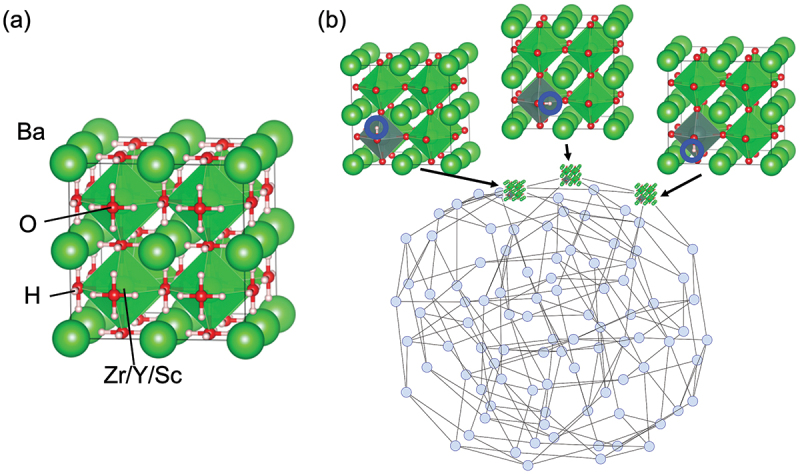
(a) Crystal structure and proton sites in 2×2×2 supercell of BaZrO3. (b) Graph used in this study for Y 12.5 at% model. Blue circles (nodes) correspond each atomic configuration, and connected black lines (edges) mean migration of proton to neighboring sites.

Here, we employed graph theory as a tool to search for viable proton diffusion pathways for non-dilute systems by considering proton–proton interaction. A graph in graph theory is a set of nodes (vertices) which are connected by edges (lines). In general, atomic configurations can be mapped to nodes on a graph and transitions between them can be treated as edges. Once a graph is constructed, reaction pathways with specified characteristics (*i.e*. that with the lowest energy barrier between two configurations) can be searched for efficiently using dynamic programming algorithms known in graph theory [205–207]. In this study, the configurations of protons were treated as nodes, and a jump to neighboring proton sites was modeled as edges in the graph. In BaZrO3 system, 96 proton sites are considered (Figure 16a), and the graph for 12.5 at% was constructed as shown in Figure 16b.

*Ab initio* calculations were performed to identify energy diagrams along proton diffusion pathways using this graph. The calculations include stable and transitional structures, the latter using the NEB method. Stable structure is the one considering proton(s) at the stable site whereas transitional structure is the saddle point between the stable structures determined by the NEB method. All the possible configurations of dopants and protons at the stable positions (96 and 4560) were considered in the 2×2×2 supercells of fully hydrated BaZrO3 with dopant concentrations of 12.5 at% and 25 at%. The former considers one dopant and one proton whereas the latter considers two dopants and two protons in the supercells. Proton diffusion pathway that possesses the lowest energy difference between stable structures was searched using the constructed graph with dynamic programming algorithm, where at least one proton moves from the most stable configuration to the same configuration in the other cell [205–207].

Descriptors important to determine structural energies were extracted using random forest regression [208]. Descriptors considered were the distance between proton and dopant, the n-th nearest neighbor cation sites from proton, O-H-O angle, B-OH-B angle, bond angle variance, Baur’s distortion index [209], and quadratic elongation [210], and the angles of diagonal vectors of dopant-O octahedra in polar coordinates, θ and ϕ. These descriptors express the atomic configuration, hydrogen bonding, octahedral distortion, and octahedral tilts. Summary statistics, such as average, maximum, minimum, and variance, of these values were used for regression analysis. If there are multiple parameters with significant correlations, one of them was kept and the rest were removed before the regression analysis to exclude redundant descriptors.

### 8.2. Decisive factors for proton diffusivity: octahedral tilts vs. distortions

**Figure 17.**
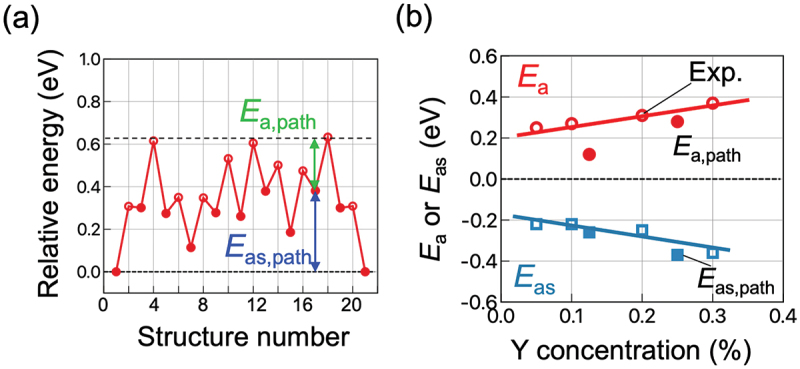
(a) Energy diagrams along obtained diffusion pathway for Y 25 at% model by graph theory and systematic *ab initio* calculations for each composition. Eas,path and Ea,path indicate the association and activation energies along the diffusion path, respectively. Stable and transitional structures were denoted as closed and open circles, respectively, where the latter was determined using the NEB method. (b) Activation and association energies for proton diffusion and their dependence on dopant concentration for Y-doped BaZrO3. Close and open circles show the calculated and experimental data, respectively.

The calculated activation and association energies of proton diffusion reproduce the experimental trend well, as shown in Figure 17. Figure 17a shows the energy diagram along the obtained pathway from graph theory analysis. In this section, the association energy along the diffusion pathway, Eas,path, was estimated as the difference in energy for proton configurations between the lowest and highest stable sites along the pathway (blue arrow), which can be considered as mobile and associated states of protons in Eq (16), respectively. The activation energy along the diffusion pathway, Ea,path, was estimated as the energy difference between the highest stable and transitional structures (green arrow). The obtained activation and association energies of 0.25 eV and 0.38 eV, respectively, at 25 at% agree with the experimentally determined values as well as their concentration dependence as shown in Figure 17b. This consistency shows that our computational models are reasonable to represent the experimental diffusivity data determined to 30 at% dopant concentration.

**Figure 18.**
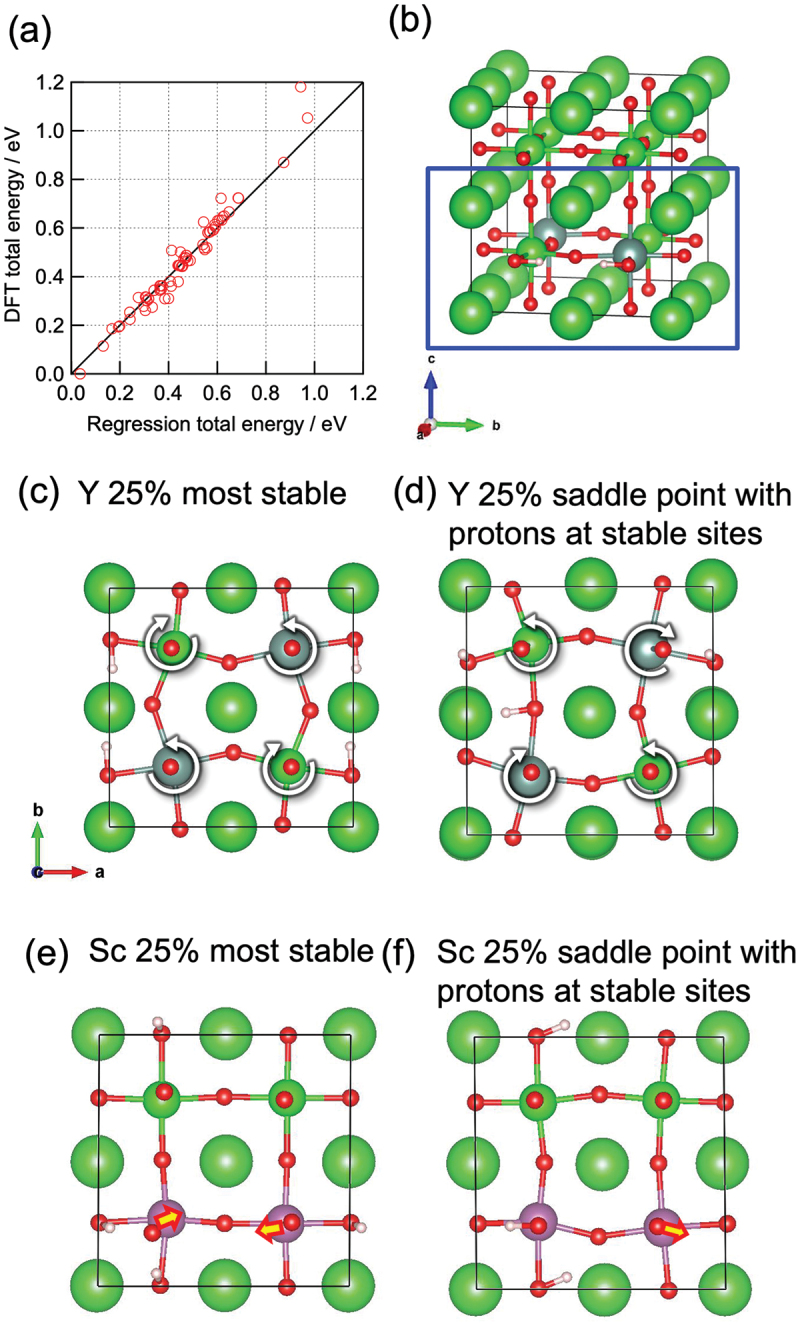
(a) Relationship between calculated and regression energies. (b) Most stable structures of Y 25 at% model. (c-f) most stable structures and the ones with the highest energy (saddle point) in the area shown by the blue box in (b) with protons at stable sites for 25 at% models focusing on the dominant structural parameters obtained from the regression results.

The regression analysis reveals that octahedral tilts and distortion (off-center displacement) are dominant descriptors in Y and Sc systems, respectively, as shown in Figure 18. The significant tilts of octahedra were found in Y-doped system (Figures 18c,d). In contrast, in Sc-doped system, the distortion (off-center) of cation is more pronounced, as shown in Figures 18e,f. The difference between Y and Sc dopants is a type of local structure, octahedral tilt or distortion that lowers the relative energy around the proton and dopant.

**Figure 19.**
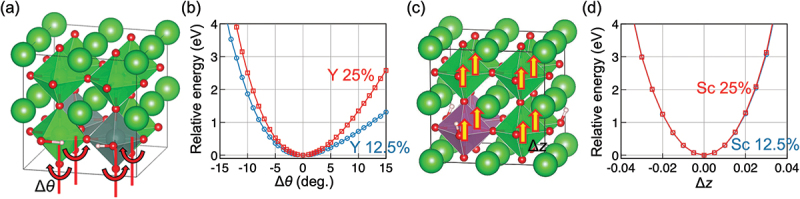
(a) Most stable structure with the octahedral tilt of Δθ for Y 25% model. (b) Relative energy dependence on difference in the octahedral tilts Δθ for most stable configurations of 12.5 at% Y model and 25 at% Y model. (c) Most stable structure with the off-center displacement of Δz for Sc 25%. (d) Relative energy dependence on difference in the off-center displacement Δz for most stable configurations of 12.5 at% Sc model and 25 at% Sc model.

The influence of octahedral tilt and distortion on its structural energy for Y and Sc, respectively, and their concentration dependence were captured in Figure 19. For Y dopant for which octahedral tilts play an important role, the lattice stiffness increases with increasing dopant content, which results in higher structural energy and thus higher activation and association energies for proton diffusion (Figures 19a,b). Such energy increase depending on dopant content was not found for Sc doping where octahedral distortion is important (Figures 19c,d), contributing to maintain high proton diffusivity in Sc-doped system. This inherent difference in structural relaxation mechanism among the dopants affects proton diffusivity and its concentration dependence. Although hydrogen bonding and proton-dopant configurations have been known as descriptors to define local structure around proton [20,22,38,99], they might be too simple to explain the dopant and concentration dependencies of proton diffusivities in the perovskite oxides.

## 9. Machine learning potentials for accelerating molecular dynamics simulation of proton diffusion with *ab initio* accuracy

As mentioned in Section 5.2, a bottleneck in applying *ab initio* MD simulations to predict proton diffusivities is the computational cost. The high cost originates from the need to solve quantum mechanical equations (usually the Kohn–Sham equations) for every instantaneous structure that appears in the simulation; this is necessary for obtaining interatomic forces, which are used to drive the dynamics of atoms based on Newton’s equation of motion. If analytical functions of atomic positions that represent interatomic forces, *i.e*. classical force fields, are available, there would be no need to solve quantum mechanical equations and the calculations can be accelerated by orders of magnitude. This would also enable natural incorporation of proton–proton correlation, temperature-dependent phase transitions, and anharmonic phonon effects that cannot be treated by the kMC method. However, parameterizing classical force fields that can accurately represent the interactions between various dopants, defects, and parent compounds, as well as covalent bond breaking and formation is a difficult task.

In recent years, ML has emerged as a promising solution to this challenge. Basically, a surrogate model is trained (fitted) to reproduce energies and interatomic forces from *ab initio* calculation and used to speed up MD simulations by several orders of magnitude. The difference from classical force fields is the use of flexible ML models (*i.e*. functions that can fit almost any data) such as neural networks instead of physics-inspired analytical functions representing Coulomb interactions, dispersion interactions, Pauli repulsion, etc. Because of the high flexibility of ML models, the amount of data (structure-energy/force relationship from *ab initio* calculation) required to fit the model is typically orders of magnitude larger than for classical force fields. This had hindered widespread use of the idea until recently, but the situation is changing rapidly with the increase in computational resources available to researchers. In addition, the development of ML models for this purpose has been very rapid in the past few years with new models reported every few months that are more accurate while requiring less training data. Here, we do not attempt a full review (see, *e.g*. [211] for some recent ones). Instead, we give a short introduction of the neural network potential (NNP) developed originally by Behler and Parinello, which is one of the earliest successful ML potentials [31,212]. Then, we discuss its application to proton diffusion in heavily Sc-doped BaZrO3.

### 9.1. Neural network potential (NNP)

**Figure 20.**
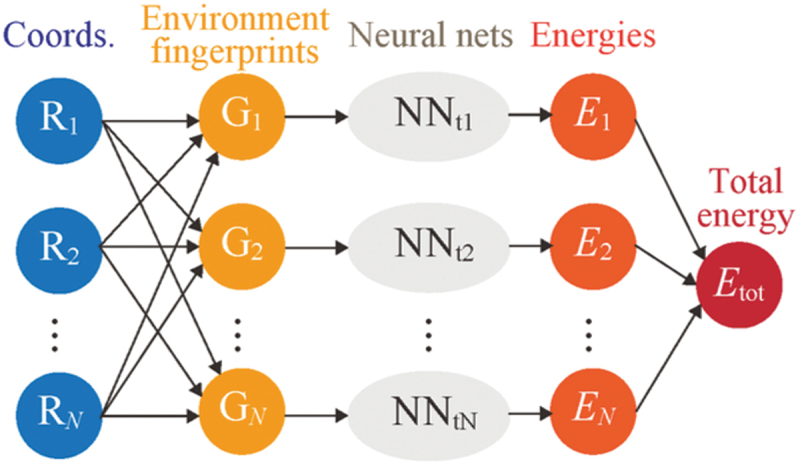
A schematic of the Behler-Parinello type neural network potential model. Reproduced from [213] under CC-BY 4.0 license.

The NNP, schematically depicted in Figure 20, relies on the ansatz that the total energy can be decomposed into a sum of atomic contributions:

(44) $$E(rN)=\sum_{i}^{\mathrm{atoms}}NNti(fi)$$

Here, NNti(fi) is a neural network model (*i.e*. the fitting function) that calculates the atomic energy for the ith atom as a function of its environment descriptor:

(45) fi=f(riRc),

which is calculated from the atomic coordinates riRc within a cutoff distance Rc from the ith atom. The same model is used for all atoms of the same species represented by ti, so the energy is invariant to permutations in the atom indices. Interatomic forces can be evaluated efficiently using the back-propagation technique to calculate derivatives of the energy with respect to atomic positions. There are many possible choices in constructing the environment descriptor; the original Behler-Parinello approach employs ‘symmetry functions’ that are invariant with respect to translation and rotation, so the model automatically respects these basic symmetries of physical systems. An issue with symmetry functions is that a combinatorial number of two- and three-body descriptors are necessary with increasing number of atomic species, and it becomes computational expensive to parametrize many-component systems (such as hydrated Sc-doped BaZrO3 with five components). To solve this issue, Artrith and coworkers proposed to use Chebyshev expansion coefficients of angular and radial distribution functions where the atomic contributions to the distribution functions are weighted differently depending on the species. This approach was used in our recent work to calculate proton diffusivities in 60 at% Sc-doped BaZrO3 [127]. In the following, we give a short review of the training and calculation process and present some preliminary data to stimulate further research.

### 9.2. Application of NNP to investigate proton diffusion

**Figure 21.**
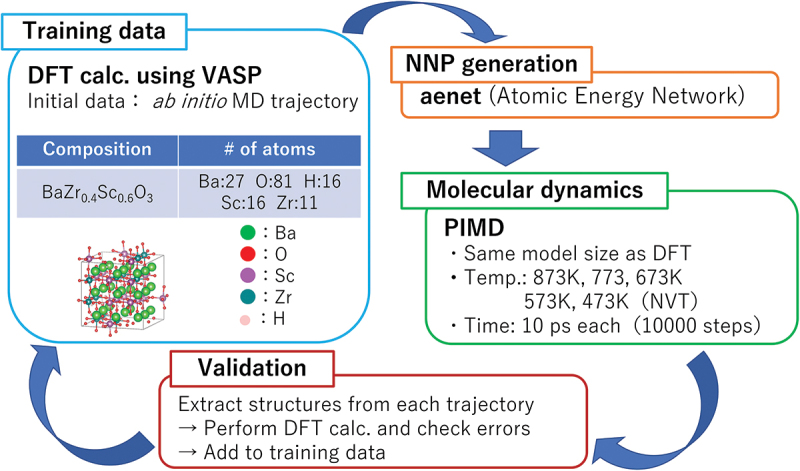
Iterative NNP training scheme used in this work. DFT stands for density functional theory.

The most difficult part of training an NNP is to accumulate a sufficiently large and well balanced training data set. For this, we employ an iterative training process as schematically shown in Figure 21. First, we perform a short *ab initio* MD simulation to accumulate the initial training data set. An NNP is trained on this initial training set, then used to perform longer NNP-MD simulations. Structures are sampled from the NNP-MD trajectory and *ab initio* calculations are performed to validate the energy predictions by the NNP. If the energy error is larger than a threshold, the structure is added to the training set and the NNP is retrained. This process is repeated until sufficient accuracy is achieved in the validation step. In this work, we employed VASP code for the *ab initio* calculations, aenet code [214] for NNP training, and NNP-MD implemented in PIMD code [215] for NNP-MD during the iterative training process. With sufficient number of cycles, we were able to generate an NNP that reproduces *ab initio* energies with a root mean squared error of less than 7 meV/atom. It is noted that the Sc positions were computed according to our lattice Monte Carlo approach detailed in Section 10.

**Figure 22.**
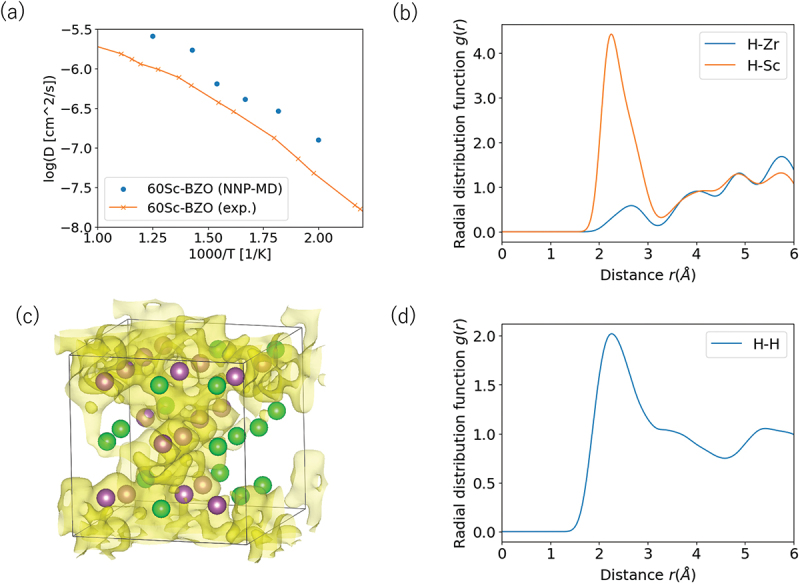
(a) Simulated and experimental [24] proton diffusivities as functions of inverse temperature, (b) H-Zr/Sc pair distribution function at 800 K, (c) concentration isosurface of protons at 800 K, and (d) H-H pair distribution function at 800 K in heavily Sc-doped BaZrO 3. The simulations were performed for deuterons then the obtained diffusivities were multiplied by $\sqrt{2}$ to compare with experimental proton diffusivities. Only Sc (purple spheres) and Zr (green spheres) are shown in (c).

This NNP was then used to perform 10 ns MD simulations of proton diffusion in 60 at% Sc-doped BaZrO3 using aenet-LAMMPS code [216,217] as a first step towards understanding how heavy Sc doping leads to fast proton conduction (Figure 3).^1^ It is noted that 10 ns is orders of magnitude longer than typical *ab initio* MD literature that report few ps to few hundred ps simulations, and this allows us to generate sufficient ion migration statistics for lower temperatures. As shown in Figure 22a, the calculated Arrhenius plot of the diffusivity reproduces the experimentally reported ‘bending’ often explained by the association effect. The proton trapping by the dopant is clearly seen in the proton/Sc and proton/Zr partial pair distribution functions shown in Figure 22b, which indicate a strong preference for protons to be trapped near Sc dopants compared to Zr of the parent lattice. However, as already suggested by previous kMC works, protons can migrate along a 3-dimensional pathway of connected dopant sites, and this is also supported by the proton concentration isosurface plot calculated from our NNP-MD trajectory as shown in Figure 22c. A notable feature of this heavily doped system is also seen in the H-H pair distribution function with a peak at the nearest neighbor site (Figure 20d), indicating a liquid-like correlation reminiscent of the prototypical superionic conductor α-AgI [218]. This reflects the fact that the proton carriers are confined to sites adjacent to Sc dopants, making the effective concentration rather high and liquid-like. We are now in the process of examining whether such correlation is favorable or detrimental to realizing fast proton conduction based on the MD trajectory.

## 10. Lattice Monte Carlo approach for thermodynamics of dopant network formation and hydration and its acceleration by machine learning

Although ML potentials have enormously expanded the applicability of MD simulations, the simulation time is still limited to 1–100 nanoseconds, depending on the calculation cell size and computer resources available. This is problematic when discussing thermodynamics of slow processes that take over seconds or even hours for equilibration such as dopant migration or proton incorporation (*i.e*. hydration). As mentioned in Section 5.2, the kMC method is also limited in this aspect. For systems with a dilute concentration of defects, the thermodynamics can be treated as explained in Section 4.1. This is because the possible atomistic configurations are limited in number, and it is often feasible to perform *ab initio* total energy calculations for all of them. For heavily doped systems, however, this is not the case, and the number can quickly skyrocket to trillions of possible configurations of dopants, oxygen vacancies, and protons. A potential solution to this problem is to use the Metropolis Monte Carlo (MMC) method, which completely ignores the kinetics to enable extremely fast equilibrium ensemble sampling. In this section, we introduce the algorithm, the technical challenges that have hindered its application to proton-conducting oxides, and how we have made such calculations feasible through the development of an original computational framework abICS (short for ab Initio Configuration Sampling toolkit) for efficient use of flagship-level supercomputers. Then, we discuss its application to successfully elucidate the local atomic configurations that activate the hydration reaction in perovskite oxides, an issue that had persisted for almost 30 years after the discovery of the first proton-conducting perovskite oxide (see also Section 3.2).

### 10.1. abICS framework for configuration sampling

#### 10.1.1. Metropolis Monte Carlo method

The MMC algorithm is a method for sampling atomistic configurations from the canonical (NVT) ensemble. It dates back to 1953, where it was applied to calculate the equation of state for rigid discs as the first instance where a computer was used for molecular simulation. The original MMC algorithm was used for canonical ensemble sampling in continuum space [219]. However, for our purposes, it is more efficient to consider sampling on a discrete lattice, as atoms in crystalline systems spend most of their time vibrating around their lattice sites. Configurations on a predefined lattice of N sites can be represented by a length-N list σN of atomic species (or vacancies) that reside on each of those sites. The ‘lattice’ MMC algorithm can be described as follows [220]:

1. Start with an initial lattice configuration σiN and calculate the *relaxed* potential energy of the configuration Ei=Erel(σiN).
2. For each iteration i,
  - Propose a trial lattice configuration σN′ by choosing a pair of atoms of different species and swapping them.
  - Calculate the *relaxed* potential energy E′=Erel(σN′) and calculate the ‘acceptance ratio’ as $p=\min[1,\exp(-\frac{E\prime -Ei}{\mathrm{kT}})]$.
  - Accept the trial configuration σN′ with probability p (or reject with probability (1−p)). When the trial configuration is accepted, set σN′→σi+1N,E′→Ei+1. When it is rejected, reuse the previous structure by setting σiN→σi+1N,Ei→Ei+1.

The algorithm basically wanders through configuration space, accepting or rejecting configurations based on ratios of temperature-dependent Boltzmann factors exp(−E/kT). Averages of physical quantities (*e.g*. energies, radial distributions, coordination numbers) and their distributions calculated from the ‘chain’ of samples {riN} are guaranteed to converge to that of the canonical ensemble for an infinite number of samples. An important property of the algorithm is the freedom of choosing ‘unphysical’ steps such as swapping of atom positions when proposing trial configurations (step 2a). This can dramatically accelerate equilibration compared to MD at the cost of losing the correct kinetics.

In principle, the energy in each trial step can be evaluated using *ab initio* calculation. In practice, however, *ab initio* calculations are too time-consuming to achieve sufficient sampling steps in a reasonable amount of time. Thus, the standard way is to fit an effective model Hamiltonian to *ab initio* calculations on a small subset of possible configurations, then to use that model for energy calculations during MMC sampling. A widely used model is the cluster expansion Hamiltonian, which expands the total relaxed energy in terms of clusters of atoms in the crystal [221–224]. The method has been used successfully to predict order-disorder phase transitions in metallic alloys with simple bcc, hcp, and fcc structures. On the other hand, many-component ionic oxides with a large concentration of defects (*e.g*. vacancy and interstitial species) pose tougher challenges, since having many components distributed over multiple sublattices causes combinatorial explosion in the number of clusters necessary to converge the expansion. In other words, it is often virtually impossible to parameterize a reliable and computationally feasible cluster expansion for the types of systems that we are interested in.

#### 10.1.2. Replica exchange sampling

**Figure 23.**
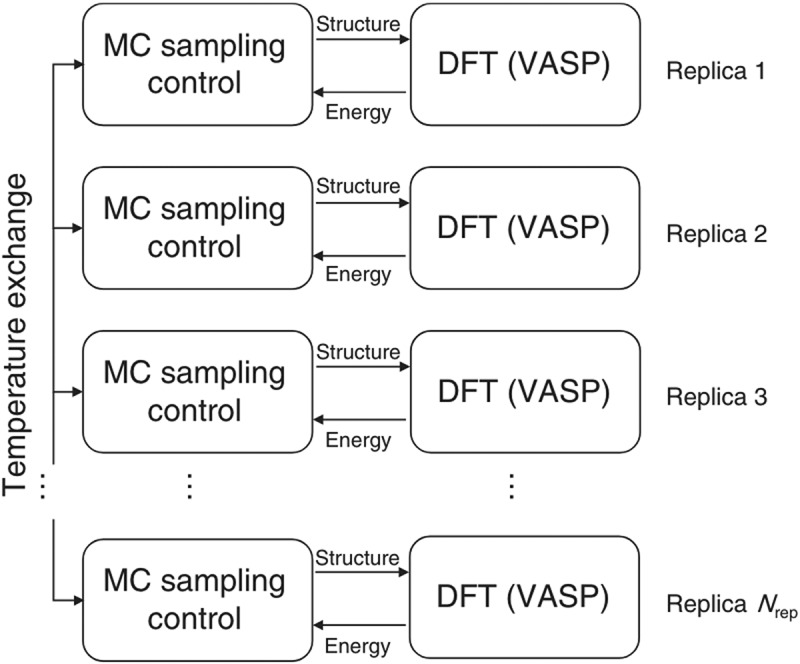
Direct coupling of RXMC sampling with *ab initio* calculations. Reproduced from [228] with permission from the Institute of Physics.

The first approach that we considered for overcoming the above issue is to improve the sampling efficiency of the MMC calculation by parallel algorithms so that direct combination with *ab inito* calculation becomes feasible. A trivial way to increase the number of samples is to run many MMC calculations in parallel with different random number seeds. However, this has limited effect in increasing the sampling efficiency at lower temperatures because the MMC algorithm often gets trapped in local minima where the acceptance ratio $p=\min[1,\exp(-\frac{E\prime -Ei}{\mathrm{kT}})]$ becomes very small for all trial configurations. The replica exchange Monte Carlo (RXMC) method [225,226] is one algorithm that was devised to overcome this issue. Essentially, it employs multiple ‘replicas’ of the simulation running at different temperatures in parallel. Low-temperature replicas with low acceptance ratios scan the region around local minima, while high-temperature replicas with higher acceptance ratios explore a more global energy landscape. When a high-temperature replica finds a new low-energy basin, it swaps temperatures with a low-temperature replica to accurately scan the immediate region for the local minimum. This alleviates the local trapping problem while sampling for multiple temperatures simultaneously. The method was first devised for spin models in the statistical physics community and later applied to speed up configuration sampling in combination with classical MD [227]. Recently, we investigated the feasibility of combining the RXMC method for the lattice configuration problem directly with *ab inito* calculation as schematically shown in Figure 23. Each of the Nrep replicas carries out lattice MC sampling independently at different temperatures, and an *ab initio* code is called to perform structural relaxation and energy calculation for each trial configuration. Temperature exchange is attempted at predetermined intervals according to Eq (46):

(46) $$p=\min\left[1,\frac{e-\beta iEje-\beta jEi}{e-\beta iEie-\beta jEj}\right]=\min\left\{1,e\left(\beta i-\beta j)(Ei-Ej\right)\right\},$$

where i,j are replica indices, β is the inverse temperature, and E is the energy of the replica. The scheme can be implemented to utilize a multi-layered parallelism of *ab initio* calculations and MC replicas, which is suited for execution on massively parallel supercomputers. This idea was first applied successfully to calculate the degree of Mg/Al site inversion in MgAl2O4 spinel as a function of temperature [228]. Later, we applied it to simulate temperature-dependent dopant/oxygen vacancy configurations for Y-doped BaZrO3 and clearly demonstrated for the first time that dopants are not arranged randomly even at very high sintering temperatures [26].

#### 10.1.3. On-lattice neural network model

**Figure 24.**
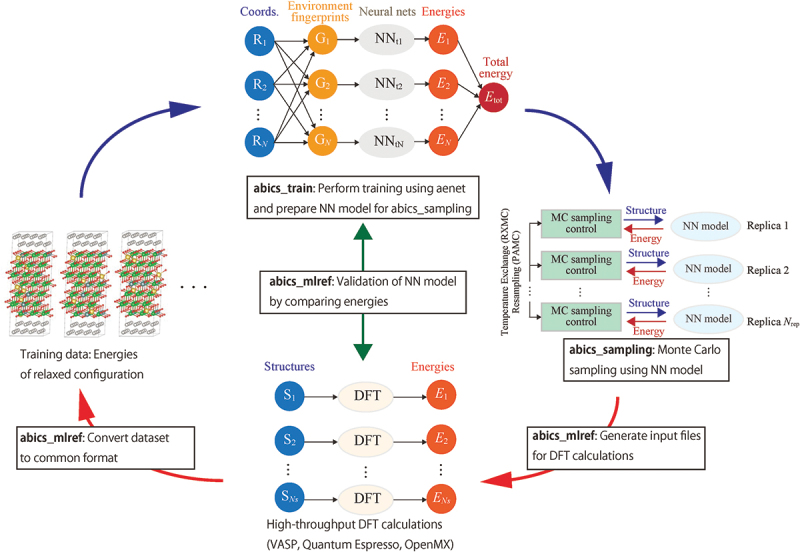
abICS framework for iterative training of neural network configuration energy models and MC sampling. Reproduced from [213] under CC-BY 4.0 license.

As described above, we were able to calculate enough samples to discuss dopant/oxygen vacancy configurations with the direct combination of *ab initio* calculations and RXMC sampling. However, the problem becomes combinatorially more challenging when considering the configuration of protons. To make such calculations feasible, we proposed to replace the *ab initio* calculation step by the NNP approach described above in an unconventional way [213]; that is, we train the same model to predict the *relaxed* energy from the coordinates of the ideal on-lattice configurations. Since the lattice MMC calculations only require the relaxed energies, this on-lattice neural network model allows us to bypass the relaxation step and accelerate the calculations by a factor of a few hundred compared to using the usual continuous coordinate NNP. Iterative training similar to that described in Section 9 for the NNP is also necessary for this on-lattice model, and we have been developing an original Python framework abICS to facilitate this process (Figure 24) [213,229]. The iterative process proceeds as follows:

1. Generate an initial set of random configurations along with input files for *ab initio* calculations.
2. Run relaxation calculations on the configurations. Different configurations can be run simultaneously, enabling efficient use of supercomputing resources.
3. Train the on-lattice neural network model.
4. Perform MMC sampling using the on-lattice model. Replica exchange and population annealing methods have been implemented to speed up the sampling using parallel computers.
5. Extract configurations from MMC sampling and perform *ab initio* calculations to validate the model. Again, configuration samples can be calculated simultaneously.
6. If the validation accuracy is not sufficient, add the configurations to the training set and repeat from 3.

With this framework, we were able to simulate the configuration thermodynamics in hydrated Sc-doped BaZrO3 and obtain temperature-dependent results that are directly comparable with experiment as we describe below.

### 10.2. Application to hydration of oxides

As noted in Section 2, the hydration reaction is the key reaction to introduce proton carriers into perovskite oxides. Although it is well understood that oxygen vacancies are thermodynamically active sites for the hydration reaction (Eq (4)), the influence of the local environment around the oxygen vacancy is poorly understood. For example, when considering the immediate neighbor cations of oxygen vacancies, there are three possible environments *M*-vO∙∙-M, *M*-vO∙∙-B, and B-vO∙∙-B. These different vacancy environments are expected to exhibit varying levels of hydration activity, but experiments have been unable to clearly discern between these environments (see Section 3.2). Recently, we applied our abICS framework and successfully resolved this issue for Sc-doped BaZrO3 [27]. Here, we give a short review of this work.

An important point to note when applying MMC sampling is that the algorithm aims to calculate the thermodynamic equilibrium configurations even when the system is unlikely to reach equilibrium under experimental conditions. For example, cation configurations are unlikely to equilibrate at temperatures where fuel cells operate or hydration reaction occurs. Thus, we employ a two-step process as follows:

1. Perform RXMC sampling of vacancy-cation configurations to obtain a representative dopant configuration [129,230] corresponding to the thermal equilibrium at the sintering temperature.
2. Fix the dopant configuration to the representative configuration at sintering temperature (∼1600 K) and perform RXMC sampling of vacancy/proton configurations with decreasing vO∙∙ and increasing proton concentrations to simulate hydration.

Such freedom to choose which degrees of freedom to equilibrate is a strength of the MMC approach and allows incorporation of prior knowledge of the kinetics. From the simulation results, we clarify the abundances of each of the vacancy environments and their activity towards hydration.

**Figure 25.**
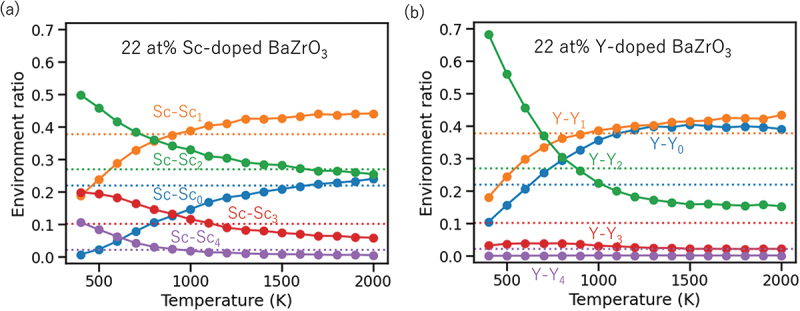
The ratio of dopant sites with given number of dopants in the nearest neighbor site in 22 at% Sc-doped (left) and Y-doped (right) systems. Dashed horizontal lines correspond to the environment ratios for a completely random dopant configuration. The data for the Y-doped system is taken from [26].

Figure 25 compares the results of step 1, *i.e*. equilibrium dopant configurations for 22 at% Sc-doped and Y-doped BaZrO3 sampled in a 3×3×3 cubic perovskite supercell. In either case, it is clear that dopants are not arranged randomly even at sintering temperature, although kMC and MMC works on ion conductors have often employed a random arrangement of dopants [23,122,126]. Also, the two dopant species result in very distinct dopant configurations. Since protons migrate along a three-dimensional network of dopants (see also Section 9), such differences in the arrangement of dopants may impact the total conductivity behavior [129,130] and should be an important ingredient in microscopic understanding of ion conductivity in heavily doped systems.

**Figure 26.**
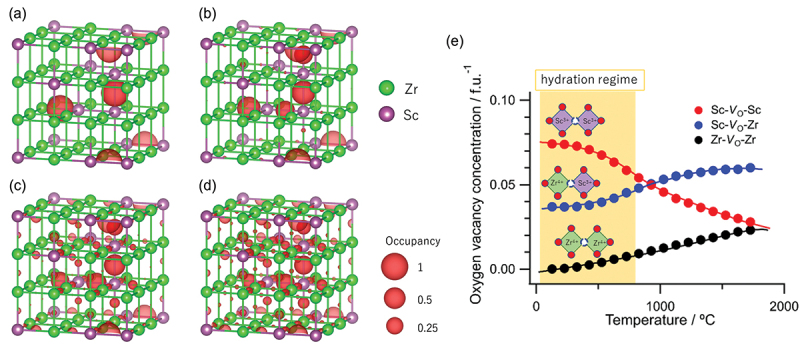
(a)-(d) visualization of oxygen vacancy occupancies at each oxygen site for varying temperatures, where the sphere size corresponds to the occupancies. Ba atoms and O atoms are omitted for clarity. (e) Ratio of oxygen vacancy environments as functions of temperature. Adapted from [27] with permission from the American Chemical Society.

In the following, we discuss the hydration thermodynamics of the Sc-doped system with the Sc arrangement fixed to that at 1600 K, *i.e*. the sintering temperature. Figures 26a–d show visualizations of vO site occupation as a function of temperature. vO are trapped between Sc dopants at low temperature and distributes more evenly as the temperature increases. The concentration of each vacancy environment is plotted versus temperature in Figure 26e, and we find that in the temperature regime for hydration, the Sc-vO-Sc environment is the most abundant followed by Sc-vO-Zr, reflecting Sc-vO association. The Zr-vO-Zr environment turns out to be very rare.

**Figure 27.**
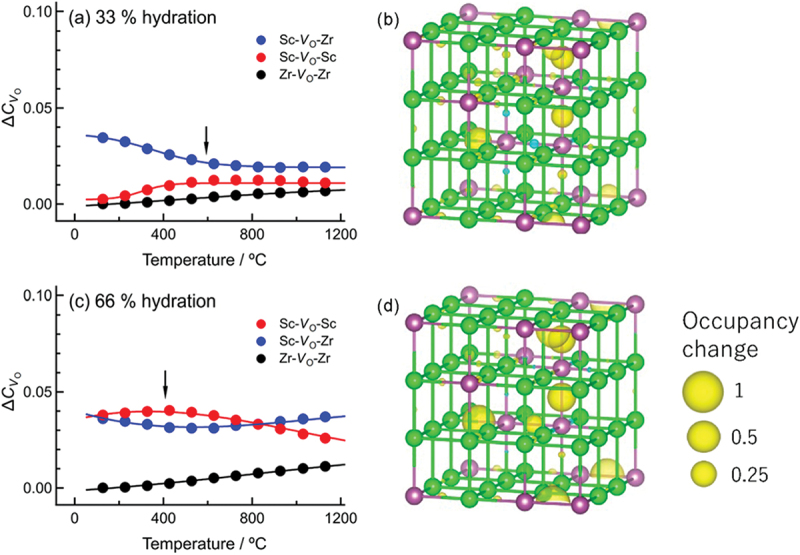
Occupancy change of oxygen vacancy sites upon hydration as a function of temperature (a,c) and that visualized in the lattice model (b,d) for 33% and 66% hydration. Reproduced from [27] with permission from the American Chemical Society.

Next, we performed RXMC sampling with increasing water content to examine which of these oxygen vacancy sites are filled preferentially upon hydration. At 33% hydration, the additional oxygen from water preferentially fills the Sc-vO-Zr sites, while Sc-vO-Sc becomes the dominant hydration site at 66% hydration (Figure 27).

**Figure 28.**
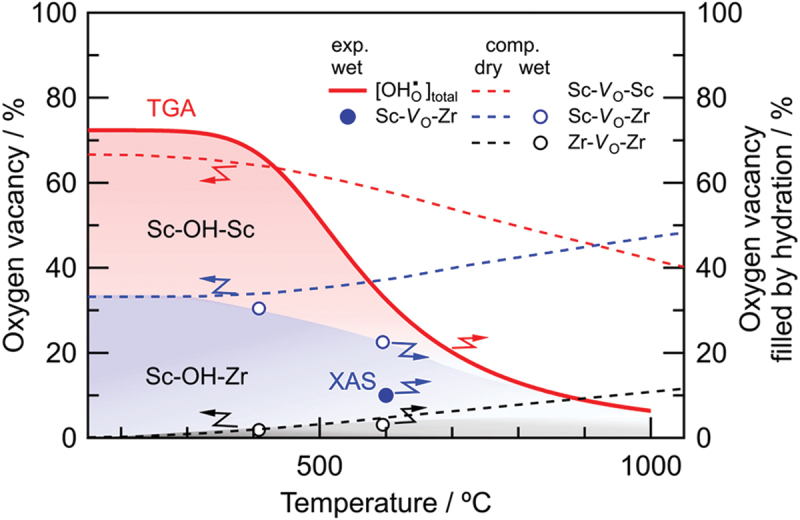
Computed and experimental ratio of oxygen vacancy configurations filled with hydroxyl groups due to hydration in 20at% Sc-doped barium zirconate combining RXMC and XAS results plotted along with experimental hydration data from thermogravimetry analysis (TGA). Reproduced from ref. [27] with permission from the American Chemical Society.

These results can be assimilated with experimental thermogravimetry data as shown in Figure 28. At higher temperature (lower hydration levels), hydration occurs preferentially on the Sc-vO-Zr site. With increasing hydration, Sc-vO-Zr sites are depleted, and Sc-vO-Sc site becomes the majority hydration site at maximum hydration level. The Zr-vO-Zr site contributes very little to hydration due to its low concentration. The computational data are consistent with in situ XAS that probe Zr and Sc coordination change upon hydration, and also with high-temperature XRD experiments that measure the chemical expansion (see Refs. [27,231] for details).

**Figure 29.**
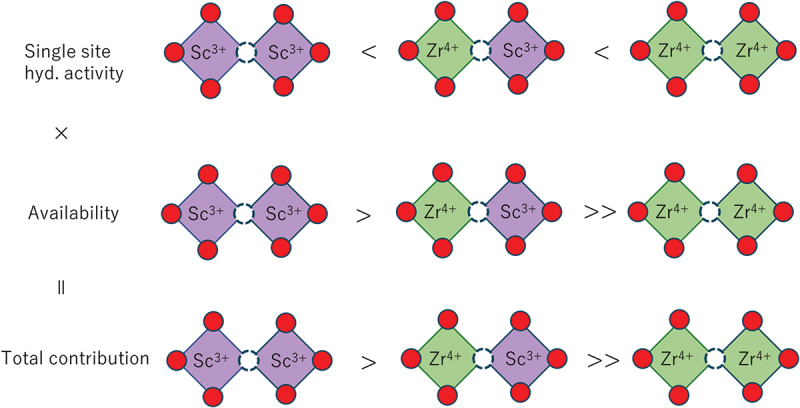
Factors contributing to the hydration activities of various vacancy environments.

Such behavior can be understood as coming from the competition between single site hydration activities and the availability (thermodynamic stability) of the vacancy environments. As shown in Figure 29, single site hydration activity is lowest for Sc-vO-Sc and highest for Zr-vO-Zr [22]. This can be understood from the relative stability of oxygen vacancies (*i.e*. the oxygen affinity discussed in Section 7): since vO is most stable when sandwiched by Sc due to association, it is least energetically favorable to fill this site upon hydration. For the same reason, thermodynamic stabilities of each of these vacancy environments are opposite to their hydration activities. In the case of 22 at% Sc-doped BaZrO3, the thermodynamic stability (*i.e*. availability) of the vacancy environments dominates the total contribution; further computation will be necessary to clarify whether this holds also for other dopants or for varying dopant concentrations. Further calculations with different dopant types/configurations should help us understand and optimize the hydration activity of doped oxides.

## 11. Conclusions

In this review, we introduced new computational and machine learning (ML) methodologies for investigating proton-conducting oxides, which were recently developed by the authors. The activation of proton conduction in oxides is attributed to point defects, specifically the formation of oxygen vacancies by acceptor doping (dopant solution), the incorporation of protons via oxygen vacancies (hydration), and subsequent proton diffusion along oxygen sublattice. Understanding the behavior of these point defects is crucial for material development, and significant experimental and computational efforts have been devoted to this area. Our methodologies are designed for accelerating material exploration based on the fundamental understanding and for gaining a deeper understanding of point defect behavior in oxides, by leveraging the accumulated knowledge in this area and insights from large-scale calculations now feasible using modern supercomputers.

In material discovery, we have addressed two new approaches. The first is the exploration of new proton-conducting perovskites by the combination of experimental database and ML technique. By designing descriptors and target variables based on the existing physicochemical understanding (structural and chemical features affecting proton thermodynamics), proton concentration was predicted with high confidence even for as-yet-reported perovskite compounds. This led to the discovery of a new proton conductor, SrSn0.8Sc0.2O3−δ. The second is the exploration of new proton-conducting *non-perovskites* by *ab initio* computational screening and interpretable ML. Basic defect calculations of hydration and dopant dissolution, which have been performed mainly for perovskites, were comprehensively applied to non-perovskite compounds in a wide structural and compositional space. ML models trained with the computational database predicted promising compounds with physicochemical interpretations, and Pb-doped Bi12SiO20 sillenate and Sr-doped Bi4Ge3O12 eulytite were discovered as new proton-conducting oxides. These are examples of how the conventional knowledge and computational framework has been extended to explore unknown structural and compositional spaces, with the use of supercomputers and ML techniques.

In understanding of point defect behaviors, we have made four attempts. The first is the demonstration of new concept, *oxygen affinity*. By taking advantages of *ab initio* calculations that can handle the fundamental processes of point defects at nanoscale, the attraction of oxygen by oxygen vacancy was calculated as oxygen affinity, which can only be assessed computationally at this time. The oxygen affinity was confirmed to be an important parameter linking hydration reactions (proton concentration) and proton trapping (proton diffusion) in BaZrO3. The second is the elucidation of complex interactions between lattice (local structure), dopants, and protons in BaZrO3. Systematic *ab initio* calculations and graph theory analysis were performed to determine representative proton diffusion paths in Y- and Sc-doped BaZrO3, and ML regression was used to investigate the structure descriptors that have the most decisive impact on proton diffusion. The results show that the manner of interactions between dopants and host lattice is significantly dependent on dopant species, with octahedral tilting for Y and off-centering for Sc. The third is the elucidation of realistic distributions of dopants, vacancies, and protons in perovskite oxides. Distributions of dopants and oxygen vacancies formed during sintering process, and the vacancy sites active for hydration can be revealed with rapid ML evaluation of lattice energies and replica exchange Monte Carlo method. The fourth is the long-time simulation of proton diffusion using accurate ML interatomic potentials trained with *ab initio* dataset. This enabled the calculation of proton diffusion spanning ∼100 ns, offering a microscopic analysis of proton trapping at intermediate temperature. The third and fourth methodologies can now be performed seamlessly to realistically handle the interactions between dopants, oxygen vacancies, and protons and to quantitatively assess their impact on thermodynamic parameters and diffusion. These are examples of how advanced calculations combined with ML techniques provide deeper insights into point defect behavior in proton-conducting oxides. These methodologies can be, in principle, applied to other systems, including non-perovskite structures, as they do not assume any specific element or crystal structure.

As described above, research on proton-conducting oxides can be greatly accelerated by using supercomputers and ML techniques, both to expand the scope of exploration into wider structural and compositional space, and to gain a deeper understanding of the behavior and interactions of point defects. Further integration of these methodologies with experiments will lead to the development of fast and stable proton-conducting oxides, contributing to the environmentally friendly electrochemical devices.

## 12. Perspective

We showed our efforts in overcoming challenges in two main areas: materials exploration and fundamental understanding of functional inorganic solids, specifically proton-conducting oxides. The first area involves addressing the limited availability of materials data and predicting functionalities in unknown compositions (extrapolation issues in ML). We practically addressed these challenges in ML by incorporating the physicochemical understanding and empirical rules of proton-conducting oxides and by generating large amount of data through computational screening. The second area is understanding and activating functions in terms of defects and their interactions in oxides. We exploited supercomputers, advanced computational and ML techniques to enable realistic analysis of the bahavior of point defects.

**Figure 30.**
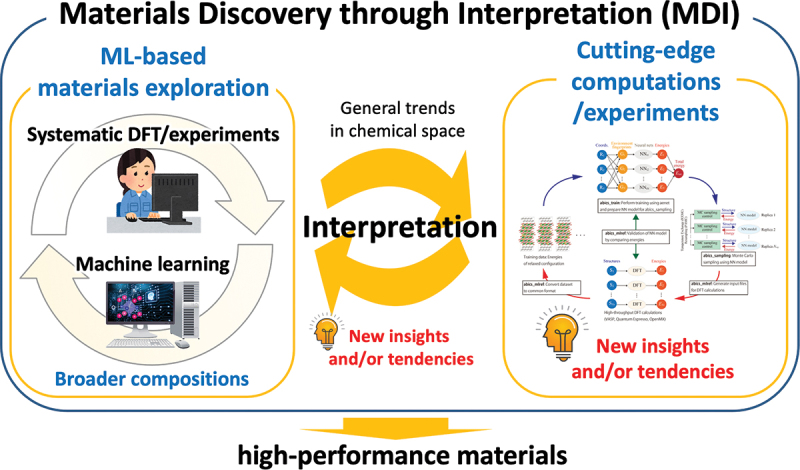
Materials discovery through interpretation. Part of this figure is reproduced from [213] under CC-BY 4.0 license.

Building on these achievements, we propose a more efficient research approach, Materials Discovery through Interpretation (MDI). This is an integration of these developed computational and ML methodologies into a sequential exploration of materials (Figure 30). The key is to incorporate our interpretations, such as new physicochemical insights, tendencies, or design principles, obtained from the cutting-edge computations/experiments to the ML model via descriptors.

We have showed such an example in Section 6.2. The addition of the physicochemical interpretation of hydration as a part of the target variable greatly enhanced the accuracy of our ML prediction although we used small, sparse training dataset. Our interpretation of high-performance proton-conducting oxides also helped to screen and identify one composition for synthesis and evaluations from thousands of candidates.

The MDI scheme also identified proton-conducting oxides with a high conductivity of 0.01 S/cm at 300  ∘C [127]. We investigated two factors to enhance the performance of rhombohedral SrSn0.8Sc0.2O3−δ (see Section 6.2): cubic symmetry (Section 3.1.4) and heavy Sc doping [24] (Section 4.3.2). For this purpose, the A-site cation was changed from Sr to the larger Ba, while the Sc dopant concentration was increased from 20 to 70at%. The resulting BaSn0.3Sc0.7O3−δ was found to be a cubic perovskite oxide that exhibits a high proton conductivity of 0.01 S/cm at 300  ∘C [127].

Although these two examples of using MDI scheme were based on descriptors known in the literature, they can be acquired through cutting-edge computations described in Sections 7-10. All new insights and trends resulting from experiments and computations have the potential to improve the MDI scheme. Integrating automated high-throughput powder synthesis methods into the MDI scheme can further accelerate materials discovery and aid in the development of high-performance functional materials specifically for electrochemical devices.

## Supplementary Material

Supplemental Material

## Note

1. We double the proton mass to enable MD calculations with longer time steps, then multiply the resulting diffusivity by $\sqrt{2}$ to account for the isotope effect [91]
